# Controlling DNA–RNA strand displacement kinetics with base distribution

**DOI:** 10.1073/pnas.2416988122

**Published:** 2025-06-06

**Authors:** Eryk J. Ratajczyk, Jonathan Bath, Petr Šulc, Jonathan P. K. Doye, Ard A. Louis, Andrew J. Turberfield

**Affiliations:** ^a^Department of Physics, Rudolf Peierls Centre for Theoretical Physics, University of Oxford, Oxford OX1 3PU, United Kingdom; ^b^Department of Physics, Clarendon Laboratory, University of Oxford, Oxford OX1 3PU, United Kingdom; ^c^Department of Physics, Kavli Institute for Nanoscience Discovery, University of Oxford, Oxford OX1 3QU, United Kingdom; ^d^School of Molecular Sciences and Center for Molecular Design and Biomimetics, The Biodesign Institute, Arizona State University, Tempe, AZ 85281; ^e^Department of Physics, Center for Biological Physics, Arizona State University, Tempe, AZ 85281; ^f^School of Natural Sciences, Department of Bioscience, Technical University Munich, Garching 85748, Germany; ^g^Department of Chemistry, Physical and Theoretical Chemistry Laboratory, University of Oxford, Oxford OX1 3QZ, United Kingdom

**Keywords:** CRISPR, strand displacement, coarse-grained model, DNA–RNA hybrids, nucleic acid

## Abstract

DNA–RNA hybrid strand displacement underpins the function of many natural and engineered systems. Understanding and controlling factors affecting DNA–RNA strand displacement reactions is necessary to enable control of processes such as CRISPR-Cas9 gene editing. By combining multiscale modeling with strand displacement experiments, we show that the distribution of bases within the displacement domain has a very strong effect on reaction kinetics, a feature unique to DNA–RNA hybrid strand displacement. Merely by redistributing bases within a displacement domain of fixed base composition, we are able to design sequences whose reaction rates span more than four orders of magnitude. We extensively characterize this effect in reactions involving the invasion of dsDNA by an RNA strand, as well as the invasion of a hybrid duplex by a DNA strand. In all-DNA strand displacement reactions, we find a predictable but relatively weak sequence dependence, confirming that DNA–RNA strand displacement permits far more thermodynamic and kinetic control than its all-DNA counterpart. We show that oxNA, a recently introduced coarse-grained model of DNA–RNA hybrids, can reproduce trends in experimentally observed reaction rates. We also develop a simple kinetic model for predicting strand displacement rates. On the basis of these results, we argue that base distribution effects may play an important role in natural R-loop formation and in the function of the guide RNAs that direct CRISPR-Cas systems.

## Significance

Important biological processes, including DNA replication, transcriptional initiation and CRISPR-Cas9 gene editing, rely on reactions in which RNA invades a DNA double helix to form a hybrid called an R-loop, or where DNA displaces the RNA inside an R-loop to create double-stranded DNA. These mechanisms are also vital in biotechnological applications such as nucleic acid sensing. Through experiments and simulations, we uncover surprisingly large variations in the rates of these displacement reactions that are due to base-pair-specific differences between DNA–DNA and DNA–RNA interaction strengths, and can be controlled simply by permuting the base sequence. Both the kinetics and thermodynamics of these hybrid strand displacement reactions can be independently fine-tuned by modifying the sequence, with potentially important consequences for biotechnological applications.

**Fig. 1.**
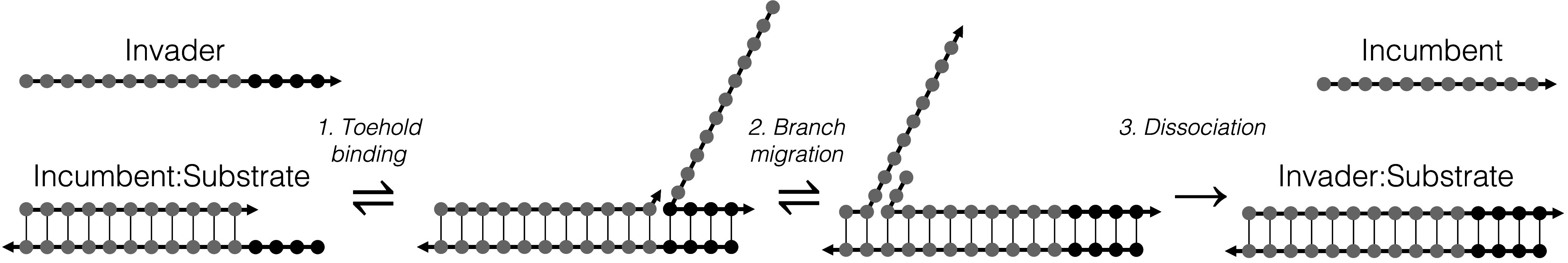
The stages of toehold-mediated strand displacement. An invader strand can bind to an unpaired portion of the substrate strand, known as a toehold. Fraying of bonds between the incumbent and substrate allows the invader to form additional base pairs with the substrate in the displacement domain, over which it competes with the incumbent, initiating a process known as branch migration which can proceed in both directions as a random walk. When there are sufficiently few base pairs between the incumbent and substrate, the incumbent can dissociate. Arrowheads indicate 3^′^ ends of strands.

Nucleic acids, with readily programmable base pairing interactions, constitute a uniquely flexible medium for directing self-organization at the nanoscale (1–5). Toehold-mediated strand displacement (TMSD)—a process in which one of the strands in an existing duplex is replaced by an invading strand (6) (Fig. 1)—is used routinely to realize dynamic and responsive self-assembled nanostructures (7, 8) as well as reaction cascades for molecular computation (9–11) and biosensing (12–14). DNA–RNA hybrid strand displacement has particular biological significance. A notable example is the R-loop, consisting of RNA annealed to one of the strands of a DNA duplex (15), which can grow or shrink by the process of strand displacement. R-loops have prominent roles in gene expression and chromatin structure, and their misregulation is associated with cancer and neurodegenerative diseases (16). DNA–RNA strand displacement also underlies the function of CRISPR-Cas endonucleases which are activated by invasion of the target dsDNA by an RNA guide to form an R-loop (17–20).

All-DNA strand displacement has been extensively characterized (21) and there are many methods for controlling its kinetics. In toehold-mediated strand displacement (Fig. 1) the invading strand initiates a strand displacement reaction (a noncovalent state transition) by binding to an unhybridized toehold. A variant is toehold exchange, where the substrate strand has toeholds at both ends to enhance the probability of the competing reverse reaction (22). Toehold length has a dramatic effect on the overall reaction rate, which increases by an order of magnitude per nucleotide up to a strong-toehold limit of 6 nucleotides (6). Toehold sequence also influences kinetics (23). Mismatches between the invader and substrate can be used to slow down strand invasion (24): This effect depends strongly on the position of the mismatch along the displacement domain (24, 25). Alternatively, mismatches between the incumbent and substrate can be used to increase the reverse rate of these reactions (26). Recently Wu et al. (27) introduced an “antitoehold,” which binds to a toehold and temporarily blocks it, enabling reversible and continuous control of strand displacement kinetics.

Strand displacement involving DNA–RNA hybrid duplexes has not been so thoroughly studied. Liu et al. (28) investigated experimentally the dependence of displacement kinetics on mismatch position, toehold length, and toehold polarity. Walbrun et al. (29) performed single-molecule force spectroscopy experiments on a limited number of hybrid strand displacement systems and concluded that base sequence plays an important role. The only comprehensive study of the effect of the displacement domain sequence on DNA–RNA strand displacement, by Smith et al. (30), showed that, for invader sequences with a high purine content, RNA invading dsDNA can be over 100-fold faster than DNA displacing RNA from a hybrid duplex. The corresponding all-DNA reaction proceeds at an intermediate rate. For low-purine sequences, the order of reaction rates is reversed. This phenomenon stems from the different relative stabilities of dsDNA and hybrid duplexes which is associated with the purine content of the invading RNA strand: Adjusting the content of purine/pyrimidine bases affects the free energy change associated with DNA–RNA strand exchange and, therefore, strand displacement kinetics. Smith et al. studied sequences in which purines and pyrimidines are distributed uniformly along each strand: The effects on strand exchange reaction kinetics of adjusting base distributions within the displacement domain, in DNA–RNA and all-DNA systems, remain unexplored.

Given the biomedical significance of DNA–RNA strand displacement, it is important to understand how to control it. In this work, which combines experiment with multiscale modeling, we propose a technique for the kinetic control of DNA–RNA toehold-mediated strand displacement that is based on rational sequence design. Our results show that it is possible to control the rates of strand displacement reactions by changing the distribution of bases in the displacement domain while keeping the overall base content identical. We demonstrate experimentally that the reaction rate can be varied by more than four orders of magnitude with almost no change to the thermodynamic drive. We also explore designs with varying base compositions. Our results are supported by simulations using oxNA (31), a coarse-grained DNA–RNA hybrid model, which reproduce the experimentally observed trends in reaction kinetics for different sequence profiles and help to understand them in terms of reaction free energy landscapes. oxNA also correctly captures the previously reported effect of average base composition on reaction kinetics (30). We introduce a simpler, less computationally intensive kinetic model for calculating reaction rates which also reproduces experimental observations, potentially facilitating the design of complex kinetic behaviors arising from differences in hybridization energies between dsDNA and DNA–RNA hybrids. For every sequence in our dataset, we also characterize an all-DNA reaction to establish a kinetic baseline: We find that the sequence dependence is much weaker, with rates varying by around one order of magnitude. Finally, we modify our kinetic model to investigate strand displacement in CRISPR-Cas9 genome editing and predict that the sequence-dependent displacement rate should strongly impact Cas9 activity, although the extent of these effects is yet to be tested experimentally.

## Results

### Principles Behind Kinetic Control Using Base Distribution.

**Fig. 2.**
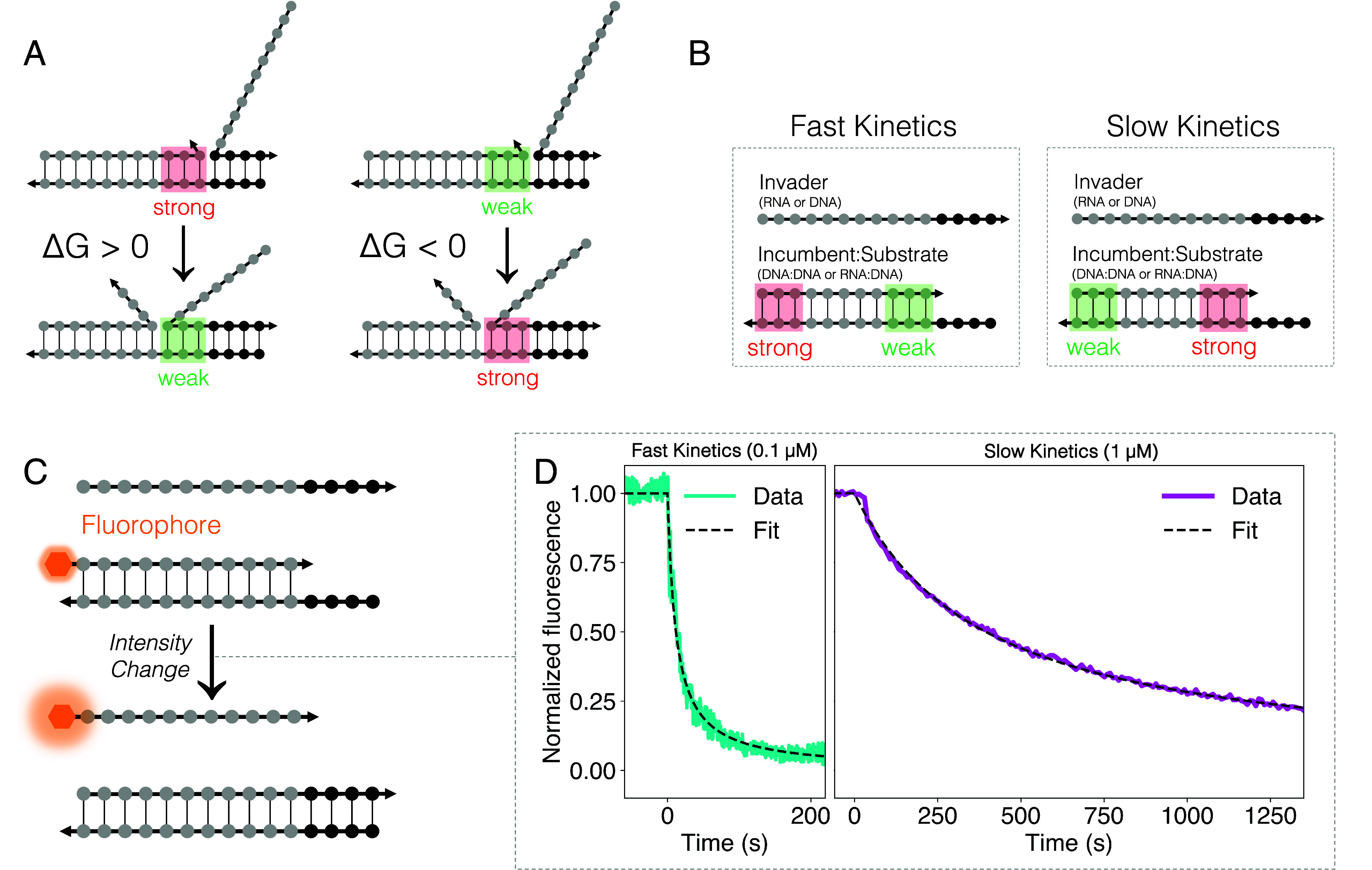
Using base distribution to control kinetics. (*A*) Differences between base pairing strengths in dsDNA and DNA–RNA hybrids with a common substrate strand are associated with local free energy changes during strand invasion, making it possible to remodel the free energy profile during strand displacement by redistributing bases along the displacement domain. (*B*) As a general design heuristic, for a given base composition of the displacement domain, rapid strand invasion is facilitated by placing incumbent-substrate base pairs that are weaker than the corresponding invader-substrate base pairs near the toehold. Base pairs that are stronger in the initial duplex create a barrier to invasion, but this has a smaller effect on reaction kinetics if placed far from the toehold. Reversing the base order slows the reaction down. (*C*) Experimental measurement of bulk reaction rates was performed by tagging the 5^′^ end of each incumbent strand with a fluorophore whose quantum yield changes when the strand is displaced. A 3^′^ quencher was attached to the substrate if the fluorophore alone did not produce a measurable signal change. Measured fluorescence traces were fitted to second-order kinetics in order to estimate rate constants. (*D*) Examples of fluorescence traces for different displacement domain sequences in reactions involving the invasion of dsDNA by an RNA strand (RNA>DNA). The fast reaction (1g in Table 1) corresponds to substrate sequence 5^′^−dTGTG (toehold)−dCCCGTTGTAAA (displacement domain)−3^′^ and the slow reaction (1a in Table 1) to a substrate with the same 5^′^ toehold but a reverse-sequence displacement domain: 5^′^−dTGTG−dAAATGTTGCCC−3^′^. In order to resolve the reaction time course, the fast reaction was carried out at a lower strand concentration.

During a DNA–RNA hybrid strand displacement reaction, DNA base pairs are replaced by hybrid DNA–RNA base pairs or vice-versa. While dsDNA and DNA–RNA hybrids have similar thermodynamic stabilities on average, the stabilities of corresponding base pairs differ significantly (32, 33). In a hybrid system, each branch migration step during strand displacement, as the invader and incumbent strands compete for binding over the displacement domain (Fig. 1), can therefore be accompanied by a substantial change in free energy. This feature—which is unique to DNA–RNA hybrid strand displacement—makes it possible to use local free energy changes to design the free energy profile of a strand displacement reaction (Fig. 2*A*).

Our sequence design is based on nearest-neighbor models for nucleic acid thermodynamics in which thermodynamic quantities associated with duplex formation are obtained by summing experimentally parameterized $\Delta G^{{}^{\circ}}$, $\Delta H^{{}^{\circ}}$, and $\Delta S^{{}^{\circ}}$ contributions for each base pair of the duplex. Here, we use $\Delta G^{{}^{\circ}}$ parameters for DNA (32) and DNA–RNA hybrids (33) to calculate sequence-dependent free energy profiles for hybrid strand displacement (*SI Appendix*, *Supplementary Note* 1).

Distributing the same bases differently along the displacement domain remodels the free energy profile of the strand displacement reaction and, as we show, modulates reaction kinetics (Fig. 2*B*). Adenines and cytosines in the DNA substrate strand are expected to cause the most significant local free energy changes as branch migration progresses: rU:dA is a weaker base pair than dT:dA [attributed to the C5 methyl group in thymine and to the location of the $2^{\prime }$-OH group in the purine (34, 35)] and rG:dC is stronger than dG:dC. We use poly-dA and poly-dC motifs in the substrate to locally raise/lower the free energy: Replacement by RNA invader sequence poly-rU of DNA incumbent poly-dT paired with poly-dA in the DNA substrate is energetically uphill, and replacement by poly-rG of poly-dG paired with DNA poly-dC is downhill (the inverse is true for DNA displacing RNA from a hybrid duplex). Substrate sequences without poly-dA or poly-dC blocks but with bases which are more evenly spread along the strand create an approximately flat free energy profile. For all of the strand displacement reactions characterized here the substrate strand was DNA with a fixed-sequence 4-base 5^′^ toehold and a displacement domain containing either 11 or 20 bases. For each displacement domain sequence, we studied the invasion of dsDNA by both RNA and DNA strands (hybrid and all-DNA strand displacement, respectively). For a subset of these sequences, we also investigated the invasion by DNA of a hybrid duplex. For sequences and chemical modifications of the strands used in each reaction see *SI Appendix*, *Supplementary Note* 2. Throughout this work we specify reactions by the displacement domain sequence of the substrate strand and the identity of the invading and incumbent strands, denoted as [invader]>[incumbent]. In all cases, the substrate strand is DNA. The three reaction types studied are RNA>DNA, DNA>RNA, and DNA>DNA.

### Experimental and Computational Measurement of Kinetics.

In order to characterize reaction kinetics experimentally, incumbent strands were tagged with a fluorophore whose quantum yield changes (36) when the strand is displaced. For a small number of reactions, the fluorophore alone did not produce a measurable signal change, and in those cases, the substrate strand was tagged with a quencher. Measured fluorescence traces were fitted to second-order kinetics to extract rate constants. We also performed experiments to validate the assumption of second-order kinetics (*SI Appendix*, *Supplementary Note* 7).

For Monte Carlo and molecular dynamics simulations of hybrid strand displacement, we use oxNA (31), a recently developed coarse-grained model for DNA–RNA hybrids. oxNA builds on the oxDNA framework (37) which has been applied successfully to all-DNA strand displacement, closely matching experimental results (6, 24–26). We use the forward flux sampling method (38) to determine the forward rate of each reaction.

We also develop a kinetic model for rate prediction, which is much less computationally costly than oxNA simulations. We adapt a model developed by Smith et al. (30) and generalize it to arbitrary sequences using nearest-neighbor parameterization of free energy changes. For more details on experimental and simulation protocols, see *Methods*.

The effect of displacement domain sequence on reaction kinetics is summarized in Table 1. We experimentally and computationally characterized a total of 30 reactions, spanning 13 different displacement domain sequences. We were able to observe rates for hybrid strand displacement reactions spanning over four orders of magnitude (sequences 3a and 3b, RNA>DNA) by changing the order of bases in the displacement domain but without changing the number of bases of each type. Kinetic differences among DNA>DNA reactions are far smaller than between hybrid reactions. oxNA simulations and kinetic model calculations are consistent with these results.

**Table 1.** Relative rate constants of strand displacement reactions studied, from experiments, oxNA simulations, and the kinetic model

	Rate constant								
	RNA>DNA			DNA>DNA			DNA>RNA		
Sequence	Experiment	oxNA	KM	Experiment	oxDNA2*	KM	Experiment	oxNA	KM
(1a) 5^′^−dAAATGTTGCCC−3^′^	1.0 ± 0.04	1.0	1.0	24 ± 1	6.8	19	100 ± 3	270	130
(1b) 5^′^−dTTGTAAACCCG−3^′^	1.7 ± 0.3	3.0	7.4	2.2 ± 0.2†	69	15	4.0 ± 0.2	22	15
(1c) 5^′^−dGAAATCGCTCT−3^′^	2.5 ± 0.1	1.3	0.46	13 ± 0.05	23	16	-	-	-
(1d) 5^′^−dGGACTACTACT−3^′^	27 ± 2	20	35	23 ± 0.4	86	15	8.9 ± 0.6	30	9.2
(1e) 5^′^−dTCTGCGAAATC−3^′^	76 ± 1	32	69	13 ± 0.1	4.0	12	-	-	-
(1f) 5^′^−dACTACCATGTG−3^′^	100 ± 4	17	87	54 ± 6	11	21	-	-	-
(1g) 5^′^−dCCCGTTGTAAA−3^′^	360 ± 30	130	176	53 ± 2	39	69	7.4 ± 0.1	2.5	1.7
(2a) 5^′^−dATGAAAATACT−3^′^	3.3 ± 0.3	9.3	3.7	8.8 ± 0.2	39	20	-	-	-
(2b) 5^′^−dTAACCCTGCCG−3^′^	76 ± 2	7.0	12	24 ± 1	11	13	-	-	-
(2c) 5^′^−dCCCTACTTCCC−3^′^	180 ± 2	165	177	94 ± 2	19	72	-	-	-
(2d) 5^′^−dTCCGTTGAAAA−3^′^	480 ± 20	81	90	75 ± 2	11	17	-	-	-
(3a) 5^′^−dGAAAAATGCTCGGTCGTCTC−3^′^	0.012 ± 0.002	-‡	0.027	4.0 ± 0.4	-	6.7	-	-	-
(3b) 5^′^−dCCCCCAGTGATAATGATGTG−3^′^	200 ± 3	-	200	35 ± 2	-	31	-	-	-

Sequences are of the displacement domain of the DNA substrate strand (the 4-base toehold dTGTG is appended to the 5^′^ end in each case). Sequences are referred to by labels shown in brackets in the *Left*-most column and have been sorted into three groups: fixed base composition (1a–1g), variable base composition (2a–2d), and longer sequences with a fixed base composition (3a, 3b). Rates are normalized relative to that of sequence 1a, RNA>DNA, for which the experimentally measured rate constant and kinetic model prediction is $k=2.6\pm 0.1\times 10^{3}$ M^−1^s^−1^. Where applicable, ranges indicate the SE of the mean.

^*^Use of the oxDNA2 model for DNA>DNA reactions is discussed in *SI Appendix*, *Supplementary Note* 4.

^†^This sequence produces secondary structure which leads to an unexpectedly low rate constant in DNA>DNA (*SI Appendix*, *Supplementary Note* 9).

^‡^The longer displacement domain in these reactions makes accurate estimation of rate constants intractable; KM = kinetic model.

### Free Energy Profiles Explain Kinetics.

The rich assortment of kinetic behaviors that we observe can be understood in terms of differences in the free energy profile, encoded by sequence, that the system needs to traverse. The dramatic ∼17,000-fold rate difference between sequences 3a and 3b for RNA>DNA is associated with the placement of the barrier to strand displacement created by the unfavorable replacement of five consecutive dT:dA base pairs with the much weaker rU:dA base pairs. The analogous all-DNA reactions differ in rate much less because base pairs are replaced like-for-like, leading to energy landscapes that are, regardless of sequence, typically much flatter.

**Fig. 3.**
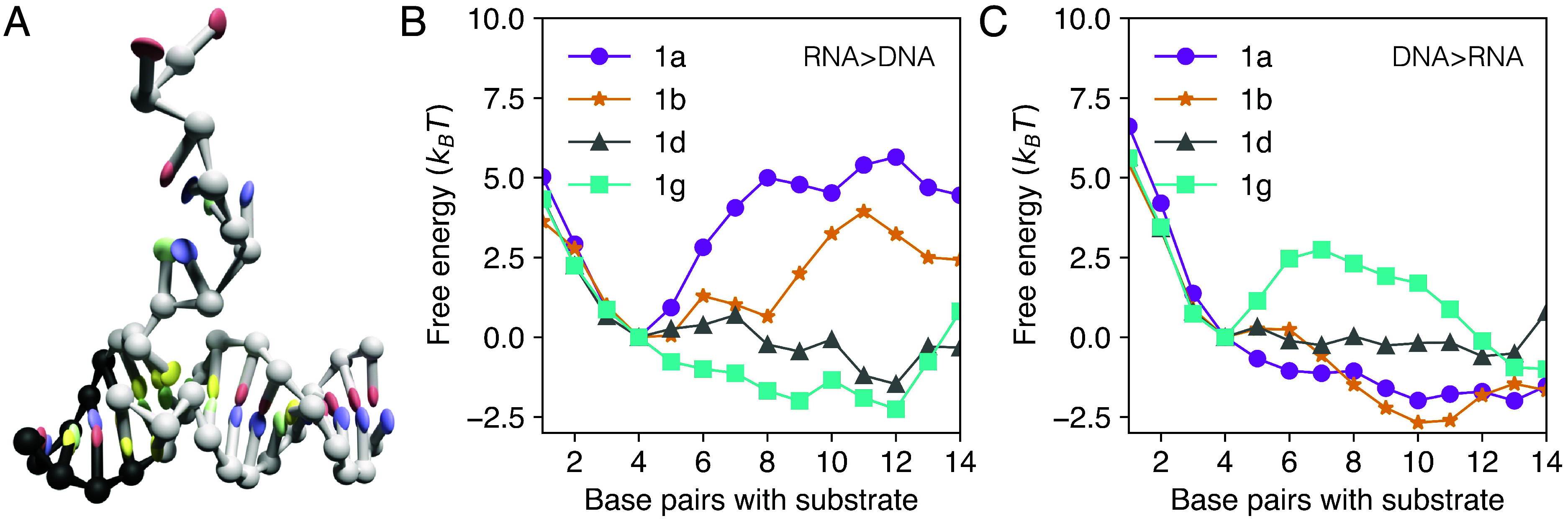
Base distribution sculpts the free energy profile of the reaction. (*A*) A snapshot from an oxNA simulation, used to extract free energy profiles. A RNA strand is displacing a DNA strand from a DNA duplex following initial binding to the exposed toehold of the DNA substrate. Computed free energy profiles as functions of the number of base pairs made between invader and substrate for four different substrate sequences: (*B*) RNA>DNA; (*C*) DNA>RNA. The first four base pairs correspond to hybridization to the toehold domain of the substrate. Free energies corresponding to 0 and 15 substrate-invader base pairs are omitted because complete dissociation of the incumbent strand is forbidden in our simulations.

Coarse-grained oxNA simulations using Virtual Move Monte Carlo (39) and umbrella sampling (40) (*Methods*) were also used to obtain the free energy profiles of strand displacement for reaction with 11-base-pair displacement domains in Table 1. Computed free energy profiles for the selected reactions are shown in Fig. 3. In both Fig. 3 *B* and *C*, the free energy profile is steeply downhill for 1 to 4 substrate-invader base pairs—this corresponds to toehold hybridization which is always favorable, since there is no competition between the incumbent and invader strands. Beyond this, the shape of the free energy profile depends strongly on the sequence within the displacement domain, which helps to explain the experimentally observed rate differences. In the most extreme case calculated, with an RNA invader, there is a 360-fold difference in displacement rate between substrate sequence 5^′^−dAAATGTTGCCC−3^′^ and its reverse (Table 1, sequences 1a and 1g). Fig. 3*B* illustrates why. For the slow reaction (pink curve), the substrate sequence dAAA adjacent to the 5^′^ toehold creates a steep initial barrier to invasion, leading to a local free energy minimum in which the system can dwell before proceeding either to complete strand displacement or, in the reverse direction, to abortive release of the toehold (both have similar free energy barrier heights). On the other hand, the fast reaction (blue curve) can progress easily after toehold binding, owing to an initially downhill free energy profile. The corresponding barrier begins at around 12 substrate-invader base pairs, but a system that has progressed to this point is more likely to complete the displacement reaction than to reverse. Sequences in which the dAAA barrier is placed centrally (orange curve) and for which the free energy profile is more nearly flat (gray curve) correspond to reactions with intermediate rates. For a DNA strand invading a DNA–RNA hybrid (Fig. 3*C*) the free energy profiles in the strand displacement region (5 to 15 base substrate-invader base pairs) are approximately inverted, so a substrate sequence that leads to rapid RNA invasion of a DNA duplex corresponds to slow DNA replacement of RNA in a hybrid duplex.

It is striking that, as the free energy profiles in Fig. 3 illustrate, net free energy changes of reactions with very different kinetics can be quite similar. Thus, permuting the base sequence in a displacement domain of fixed composition can allow for kinetic control independent of thermodynamic drive—we discuss this in more detail below.

### oxNA Reproduces Kinetics of Toehold Exchange Reactions.

**Fig. 4.**
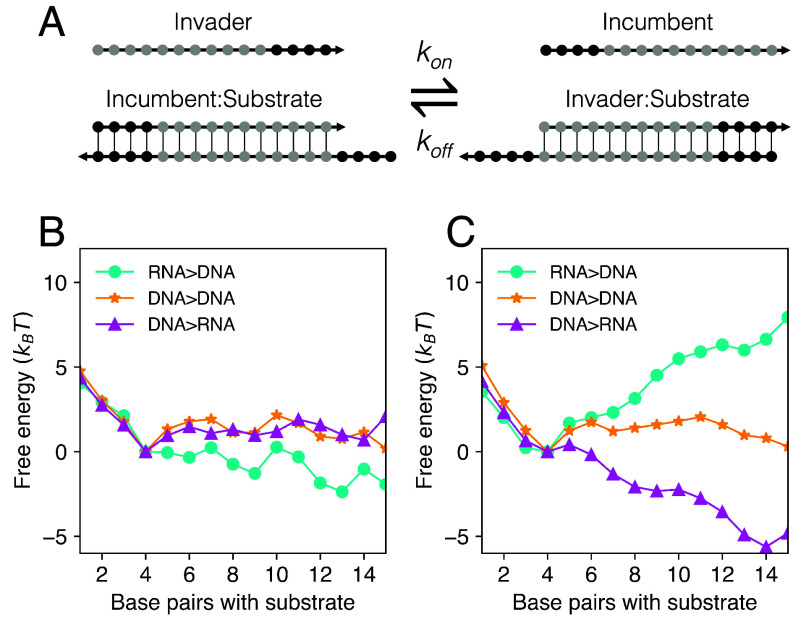
Free energy profiles of toehold exchange reactions. (*A*) In a toehold exchange reaction, the substrate strand has two toeholds, meaning that, following successful invasion, the incumbent strand can rebind and initiate a reverse reaction. Free energy profiles of selected reactions from Smith et al. (30) (*SI Appendix*, *Supplementary Note* 2) derived from oxNA simulations for systems with (*B*) high-purine and (*C*) low-purine displacement domains in the invading strand.

In order to better isolate the effects of sequence on hybrid strand exchange we have restricted our experiments to strand displacement reactions driven by a single toehold, initially single-stranded, which is fully hybridized in the product duplex. However, a toehold exchange mechanism, illustrated in Fig. 4*A*, is often used in strand displacement systems to enable reversibility and to allow finer kinetic control. DNA–RNA hybrid strand displacement in toehold exchange reactions was studied by Smith et al. (30). In order to further benchmark oxNA as a tool for predicting sequence-dependent effects in strand exchange, we have performed additional simulations of toehold exchange reactions.

To economize on computational cost, we simulated a subset of the reactions characterized by Smith et al., those with the shortest strand lengths. Free energy profiles and estimates of relative reaction rates were obtained as described previously (*Methods*). In all cases, the substrate strand is DNA: We simulated RNA>DNA, DNA>DNA, and DNA>RNA. For each of these systems, the effect of changing the purine content of the invading strand’s displacement domain is considered (for base sequences see *SI Appendix*, *Supplementary Note* 2). Experimental reaction rates measured by Smith et al. (30) and estimates from oxNA simulations and kinetic model calculations are recorded in Table 2; accompanying free energy profiles for each reaction are in Fig. 4. Again, oxNA reproduces trends in reaction rates, providing a semiquantitative fit for most of the reactions. The most significant deviation between simulated and experimental rates can be observed for the case of low-purine RNA>DNA which we attribute to underestimation of the strength of rU:dA base pairs by oxNA (see *SI Appendix*, *Supplementary Note* 4 for a more detailed discussion). Free energy profiles reveal the reason for rate differences—slower reactions are energetically uphill.

**Table 2.** Relative experimental rate constants for selected toehold exchange reactions from Smith et al. (30) (*SI Appendix*, *Supplementary Note* 2) compared to oxNA simulations and the kinetic model

	Rate constant								
	RNA>DNA			DNA>DNA			DNA>RNA		
Sequence	Experiment	oxNA	KM	Experiment	oxNA	KM	Experiment	oxNA	KM
High purine	180	290	300	33	18	49	1.0	1.0	1.9
Low purine	21	0.1	40	99	30	62	370	400	71

Rates of experimental and oxNA-simulated reactions are normalized relative to those for high-purine DNA displacing RNA from a hybrid duplex (*Top Right*), for which the experimental rate constant is $k=5.8\times 10^{2}$ M^−1^s^−1^. For kinetic model results the scale factor is as in Table 1. Labels “high purine” and “low purine” used by Smith et al. (30) refer to the composition of the displacement domain of the invading strand.

### Exploring the Thermodynamic and Kinetic Design Space.

While oxNA makes it possible to compute displacement rates, the required simulations are computationally costly, typically requiring several days using 20 CPUs. Here, we introduce a simple kinetic model for hybrid strand displacement, enabling rapid estimation of rate constants. Our kinetic model is adapted from Smith et al. (30), which is based on a model for DNA strand displacement introduced by Srinivas et al. (6). Strand displacement is modeled as a one-dimensional random walk along a Markov chain of states, starting with the invading strand unbound in solution, followed by progressive (nucleotide-by-nucleotide) toehold binding and branch migration, and ending with incumbent dissociation. The model is parameterized by a single rate constant (corresponding to the formation of a base pair in the toehold), which sets the time scale for all transitions between states, and by free energy changes corresponding to different physical processes throughout the reaction (*Methods* and *SI Appendix*, *Supplementary Note* 1). We extend the model of Smith et al. (30) to include a sequence-dependent activation barrier for each branch migration step as the invader and incumbent compete for base pairing to the substrate. We introduce parameter $\Delta G_{\mathrm{rd}}\left(s,n\right)$, derived from nearest-neighbor models (32, 33), corresponding to the sequence-dependent free energy change on replacement of the nth base of the DNA incumbent in displacement domain s by an RNA invader. The thermodynamic drive for the hybrid strand displacement reaction (without the contribution from the toehold) is $\Delta G_{\mathrm{RD}}\left(s\right)=\sum_{n}\Delta G_{\mathrm{rd}}\left(s,n\right)$. In the case of DNA>RNA, the corresponding parameter is $-\Delta G_{\mathrm{rd}}\left(s,n\right)$. We calculate an analogous quantity for DNA>DNA reactions, $\Delta G_{\mathrm{dd}}\left(s,n\right)$, using nearest-neighbor coaxial stacking (41).

For the TMSD reactions recorded in Table 1 the experimentally observed trend in rates is reproduced by the kinetic model. While the model should not be relied on for quantitative prediction of reaction rates, we expect it to provide biophysical insights and to be a useful tool for sequence design.

**Fig. 5.**
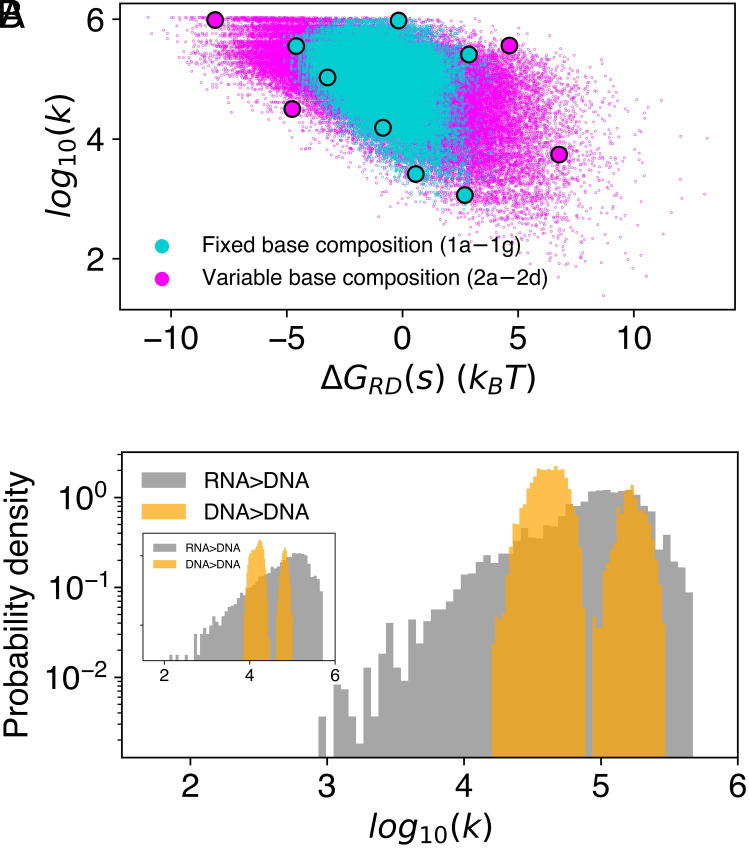
Exploring the design space of random sequence pools using the kinetic model. (*A*) Scatter plot of computed reaction rates k and net free energy changes $\Delta G_{\mathrm{RD}}\left(s\right)$ for invasion of a DNA duplex mediated by a 4-base toehold. Blue points correspond to RNA invaders with the same fixed base composition as in the experiments (sequences 1a–1g) reported in Table 1. Pink points correspond to RNA invaders with arbitrary base compositions. The eleven displacement domain sequences characterized experimentally are highlighted. Both displacement domain libraries (fixed and variable base composition) comprise $10^{5}$ random sequences. (*B*) Rate distributions of the same fixed-base-composition random library as above, comparing the invasion of dsDNA by RNA and by DNA. The *Inset* shows an analogous calculation for $10^{5}$ sequences of length 20, with a fixed, uniform base distribution. All rates are given in units of M^−1^ s^−1^.

We apply the kinetic model to random sequence pools to explore the design space for RNA invading a DNA duplex by TMSD. Fig. 5*A* shows predicted results for a selection of the reactions characterized in Table 1 and for a library of $10^{5}$ sequences with a 4-base toehold and random-sequence 11-base displacement domain. These results indicate that the kinetics and thermodynamics of hybrid strand displacement can be tuned independently over wide ranges. For sequences with a fixed base composition (blue points), the variation in duplex stability is due to nearest-neighbor effects. Lifting the base composition restriction (pink points) expands the sequence design space even further. We emphasize that the sequence-dependent tuning of reaction kinetics and thermodynamics over such wide ranges is only possible in hybrid strand displacement systems in which the change in free energy on replacing a DNA–DNA base pair with a RNA–DNA base pair can be used to control reaction free energies and rates. The contrast between RNA>DNA and DNA>DNA strand displacement is highlighted by Fig. 5*B*, which shows probability distributions of reaction rates for random sequences. As the *Inset* illustrates, extending the displacement domain extends the range of reaction rates; longer sequences permit longer poly-rU blocks, allowing for higher possible energy barriers. The bimodality in the DNA>DNA rate distribution is discussed in *SI Appendix*, *Supplementary Note* 1.

Similar calculations using different toehold lengths shows that, in the strong toehold limit of 6 bases, the rate distribution narrows: Kinetic control using base distribution is most effective below the strong toehold limit (*SI Appendix*, *Supplementary Note* 6).

### Toward Modeling CRISPR-Cas9.

DNA–RNA hybrid strand displacement is an integral part of the mechanism of CRISPR-Cas gene editing, in which the nontarget strand of the target DNA duplex is displaced by an RNA invader. Mismatches between guide and target can slow down or even abolish the catalytic activity of Cas9 (42–45). As we have shown, base sequence is a strong determinant of displacement kinetics in DNA–RNA hybrid TMSD; we would also expect it to influence the activity of CRISPR-Cas systems, even for perfectly matched guide and target sequences (46). Other factors that are known to influence Cas9 activity include the multiplicity of on-target and off-target sites, PAM sequence, secondary structure in the guide RNA (47), target site flanking sequence (46), DNA supercoiling (48), and many others (49). In the analysis below we ignore these and focus only on R-loop formation.

**Fig. 6.**
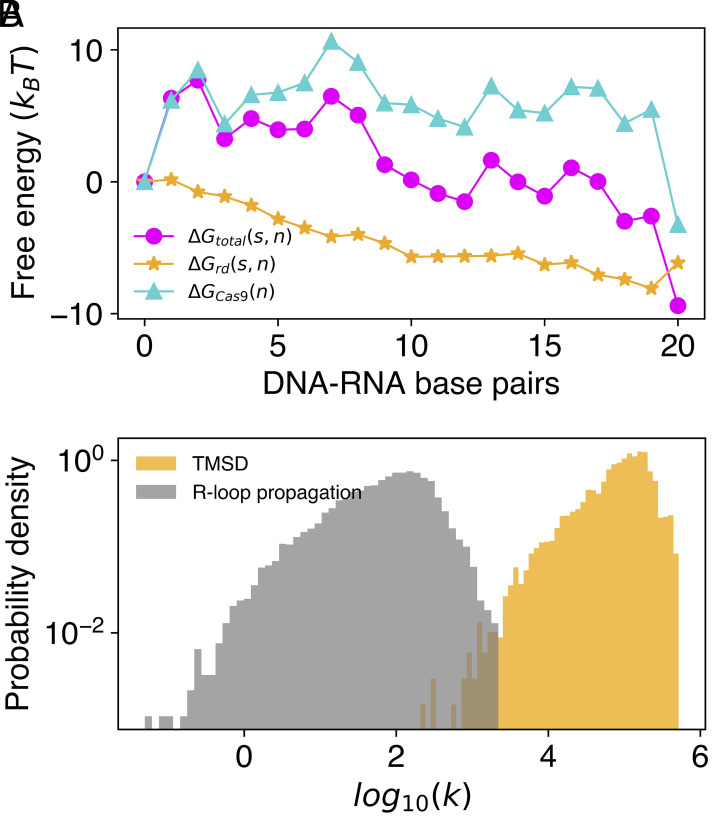
Investigation of sequence-dependent R-loop formation kinetics in the context of CRISPR-Cas9. (*A*) Components of the R-loop propagation free energy in CRISPR-Cas9. $\Delta G_{\;\text{total}}\left(s,n\right)$ is the local change in free energy, corresponding to R-loop elongation by one base pair, estimated by Eslami-Mossallam et al. (50) for one particular target sequence s. $\Delta G_{\mathrm{rd}}\left(s,n\right)$ is the contribution to $\Delta G_{\;\text{total}}\left(s,n\right)$ due to differences in hybridization free energies between dsDNA and the hybrid duplex. The difference between these quantities is used to estimate a sequence-independent, position-dependent component to the overall free energy, $\Delta G_{Cas9}\left(n\right)$, arising from protein-nucleic acid interactions. (*B*) Rate distributions for regular toehold-mediated strand displacement and for a simple model of R-loop propagation which is approximated by adding $\Delta G_{Cas9}\left(n\right)$ to $\Delta G_{\mathrm{rd}}\left(s,n\right)$ to generate the free energy profile for strand invasion for any target sequence. A random pool of $10^{5}$ sequences with uniform base content (4 bases in toehold, 20 in displacement domain) was used to generate the data shown. Rates are given in units of M^−1^s^−1^.

In order to explore potential effects of target base sequence on CRISPR-Cas9 activity, we make a simple modification to our kinetic model to better mimic strand displacement as it happens in the constrained environment provided by binding to Cas9. We assume that the free energy change at displacement step n during R-loop formation—a process involving the displacement of a DNA strand within a duplex by RNA, to produce a DNA–RNA hybrid—takes the form $\Delta G_{\;\text{total}}\left(s,n\right)=\Delta G_{\mathrm{rd}}\left(s,n\right)+\Delta G_{Cas9}\left(n\right)$, where $\Delta G_{\mathrm{rd}}\left(s,n\right)$ is the sequence-dependent free energy change discussed above and $\Delta G_{Cas9}\left(n\right)$ is intended to capture effects of protein-nucleic acid interactions and other contributions to the free energy which are assumed to be sequence-independent. Eslami-Mossallam et al. (50) developed a kinetic model for predicting off-target activity in CRISPR-Cas9 which includes the free energy changes during R-loop formation as fitting parameters, providing an effective free energy landscape equivalent to $\Delta G_{\;\text{total}}\left(s,n\right)$. Their model was developed using a particular target sequence 5^′^−dGACGCATAAAGATGAGACGCTGG−3^′^. Using these data, we can estimate $\Delta G_{Cas9}\left(n\right)$ as $\Delta G_{\;\text{total}}\left(s,n\right)-\Delta G_{\mathrm{rd}}\left(s,n\right)$, as shown in Fig. 6*A*. We incorporate this correction to the free energy into our kinetic model and investigate how it affects the distribution of rates, for RNA invading a perfectly matched DNA duplex during the formation of an R-loop, for a random pool of sequences. Fig. 6*B* shows that this modified free energy landscape produces rates which are even more strongly sequence-dependent than for toehold-mediated strand displacement and are thus broadly tunable (reasons for this are discussed in *SI Appendix*, *Supplementary Note* 8). Reliable quantitative prediction of the effects of target sequence on Cas9-mediated R-loop formation would require much more extensive experimental parameterization, but the conclusion that the invasion rate is expected to be strongly sequence dependent is robust. This suggests the possibility of understanding, and designing, on-target CRISPR-Cas9 activity by considering hybridization energetics during R-loop formation. If Cas9-mediated R-loop formation is strongly sequence-dependent, which up to this point, has generally been assumed to not be the case (47), it could lead to a better mechanistic understanding of both on- and off-target activity. In applications such as CRISPR-induced gene knockout, for which the range of possible target sites is broad (51), the base distribution within candidate target sites could inform sgRNA design. Future work will focus on modeling sequence-dependent effects more rigorously, including the effects of mismatches in different sequence contexts.

## Discussion

Our results demonstrate that the significant differences in stability between corresponding RNA–DNA and DNA–DNA base pairs provides opportunity to tune both the rate and the thermodynamic driving force of a hybrid strand displacement reaction. By strategically positioning base pairs that favor or disfavor substitution of a DNA base with the corresponding RNA base, the free energy profile of a strand displacement reaction can be tuned without significantly affecting the overall free energy change of the reaction. The thermodynamic driving force for the reaction can be tuned by changing the base composition of the displacement domain. It can also be changed, without changing base composition, by making use of the effect of neighboring base pairs on the free energy change on base substitution. This is a much richer design space than DNA–DNA strand displacement in which, unless mismatched bases are introduced, base pairs are replaced like-for-like and each step in the competition between incumbent and invader strand is approximately energetically neutral. Our results have also provided insight into how the free energy profile of a process affects its kinetics. Processes with higher barriers, regardless of the exact shape of the profile, are typically slower. If barrier heights are equal, the further the barrier from the toehold, the faster the kinetics.

We have tested these principles by demonstrating toehold-mediated strand displacement reactions that are rationally designed to tune reaction rate without changing base composition. Earlier work on toehold exchange reactions showed the effects of altering base composition (30), which we further validated in the context of TMSD on newly designed sequences. Coarse-grained simulation of hybridization reactions using oxNA (31) reinforces our mechanistic understanding of these effects and provides a semiquantitative tool for the design of base sequences to obtain desired kinetic behaviors.

The simpler Markov chain kinetic model of strand displacement also reproduces the observed effects of base sequence on strand displacement rates. It can be used to explore a much wider sequence space, revealing the broad ranges over which reaction rates and driving forces can be tuned. A very approximate extension to the kinetic model based on estimates of the free energies associated with the Cas9-mediated formation of an R-loop between RNA guide and target DNA duplex (50) indicates that, even in the case of a perfectly matched guide RNA, R-loop formation rates may be strongly dependent on target sequence. The sequence context of a mismatch with the target could also be important. We intend to explore this phenomenon further by developing a kinetic model for CRISPR-Cas9 gene editing that incorporates the effects of target sequence.

## Methods

### Oligonucleotides.

NUPACK (52) was used for sequence design to minimize unwanted interactions including secondary structure and dimerization—more details are provided in *SI Appendix*, *Supplementary Note* 9. Full sequences and their chemical modifications are available in *SI Appendix*, *Supplementary Note* 2. DNA and RNA oligonucleotides were purchased from Integrated DNA Technologies. Incumbent strands were chemically modified with 6-FAM (fluorescein) at the 5^′^ end. In a small number of reactions, we used substrate strands with a black hole quencher (BHQ1) at the 3^′^ end in order to produce a measurable fluorescence change upon addition of the invader. Strands were ordered with standard desalting on a 100 nM scale. Each strand was resuspended in TE buffer (10 mM Tris-HCl, 1 mM EDTA, pH 8) to a concentration of 100 µM and stored at 4 ^°^C away from light.

### Fluorimetry Experiments.

All reactions were carried out at 20 ^°^C in a buffer containing 10 mM Tris-HCl pH 8, 1 mM EDTA, and 10 mM MgCl_2_. Before each measurement, substrate and incumbent strands were mixed in equal amounts and left for 10 min to hybridize. 148.5 µL of the substrate-incumbent mixture was added to the cuvette and a baseline measurement performed for at least 1 min, followed by addition of 1.5 µL of invader strand solution. For most reactions, measurement was conducted at a final strand concentration of 1 µM. Exceptions were sequence 1a, DNA>RNA and sequence 1g, RNA>DNA, conducted at 0.1 µM, and also sequence 3a, RNA>DNA, which was conducted at 0.5 µM. Each measurement was repeated three times (*SI Appendix*, *Supplementary Note* 3).

### Analysis of Fluorescence Data.

During single-cuvette measurements large intensity fluctuations at around $t_{0}$, caused by the insertion of the pipette tip into the cuvette, were removed from the raw data using the Hampel filter in Python3.

To obtain estimates of rate constants, we assumed second-order kinetics, an assumption validated experimentally (*SI Appendix*, *Supplementary Note* 7). Fluorescence traces were fitted to the function

$$\begin{array}{c} F\left(t\right)=\left\{\begin{array}{cc} F_{0}, & t\leq t_{0}\\ F_{\infty }+\frac{F_{0}-F_{\infty }}{1+kc(t-t_{0})}, & t>t_{0}, \end{array}\right. \end{array}$$

where $F_{0}$, $F_{\infty }$ are the intensities before addition of the invader and at t=∞, respectively, $t_{0}$ is the time at which the invader strand is added, k is the rate constant and c is the initial concentration of each strand. Fitting was carried out using optimize.curve_fit from the SciPy library (53), with $F_{0}$, $F_{\infty }$, $t_{0}$, and (kc) as free fitting parameters.

The normalized fluorescence intensity plotted in Fig. 2*D* was calculated as $F_{\;\text{norm}}\left(t\right)=\frac{F\left(t\right)-F_{\infty }}{F_{0}-F_{\infty }}$, where F(t) is the measured intensity.

### Simulations with oxNA.

All simulations were performed using the oxNA coarse-grained model (31) which combines the most recent versions of oxDNA (54) and oxRNA (55, 56). The simulation temperature was 25 ^°^C. Monovalent salt concentration was set to 0.5 M for all forward flux sampling simulations and to 1 M for Virtual Move Monte Carlo simulations. We do not expect a significant difference between results obtained at these two salt concentrations.

Reaction rate constants were estimated by running molecular dynamics simulations coupled with forward flux sampling (38). We used a Brownian thermostat with the time step set to 0.005 simulation units. Brute-force simulation of a full strand displacement event is generally intractable: In a forward flux sampling scheme, the reaction coordinate is partitioned by checkpoints known as interfaces and the full reaction trajectory pieced together from simulations of transitions between interfaces. Each simulation is initiated with an invader and a substrate-incumbent complex in a cubic simulation box and equilibrated for $10^{5}$ time steps. The box side length was set to 13.6 nm for TMSD reactions presented in Table 1 and to 25.5 nm for the toehold exchange reactions presented in Table 2. By running several simulations, the rate at which the first interface is reached can be estimated. Similarly, for subsequent interfaces, we compute the probability of reaching an interface starting from the one before it. An effective rate can then be estimated as $k=\Phi_{0}\prod_{i=1}p_{i}$, where $\Phi_{0}$ is the flux across the first interface and $\left\{p_{i}\right\}$ are subsequent crossing probabilities. Note that the absolute values of these rate constants depend on the details of the simulations; only ratios between calculated rates are physically significant. Full details of the chosen interfaces for every reaction simulated, and all crossing probabilities, can be found in *SI Appendix*, *Supplementary Note* 4. Absolute rate constants of the reference reactions in Tables 1 and 2 can be found in *SI Appendix*, *Supplementary Note* 3.

Free energy reaction profiles were calculated using the Virtual Move Monte Carlo algorithm (39) in which clusters of particles which move in a similar fashion are selected and then randomly translated or rotated in order to explore conformational space more efficiently. Umbrella sampling (40) was also used to flatten the free energy profile by introducing weights to augment acceptance probabilities of Monte Carlo moves to high-energy states. We chose a 2-dimensional order parameter consisting of the numbers of substrate-invader and substrate-incumbent base pairs. Umbrella sampling weights were chosen to depend only on the number of substrate-invader base pairs and were adjusted such that the biased populations of these sets of states are within an order of magnitude of each other. For a state with a given number of substrate-invader base pairs, the exact positions of the base pairs along the strand are not constrained, although in most states these form a contiguous, fully hybridized sequence starting from the toehold. Intrastrand interactions are allowed, but we expect them to be minimal, as sequences are designed to be free of secondary structure. In order to accelerate sampling we forbid strand dissociation. For each displacement reaction, we ran 10 independent simulations for a total of at least $4\times 10^{9}$ Monte Carlo steps. The free energies of states were obtained from the unbiased population probabilities using the relation $p\left(G_{i}\right)\propto e^{-G_{i}/k_{B}T}$. The free energy of a fully bound toehold was set to the reference value of G=0.

### Kinetic Model Parameterization.

Our kinetic model (Table 3) is adapted from Smith et al. (30), using the same values for many of the model’s parameters (*SI Appendix*, *Supplementary Note* 5). The model treats strand displacement as a 1D Markov chain, with states being defined by the number of consecutive base pairs formed by the invader, starting with the invader unbound in solution. We assume that only transitions between adjacent states are possible. A new parameter required to model DNA–RNA strand exchange is $\Delta G_{\mathrm{rd}}\left(s,n\right)$, the local sequence-dependent free energy change during RNA>DNA branch migration, where s is the sequence and n the position along the displacement domain. For DNA>RNA we use $-\Delta G_{\mathrm{rd}}\left(s,n\right)$. This and the corresponding parameter $\Delta G_{\mathrm{dd}}\left(s,n\right)$ for DNA–DNA strand exchange are computed using nearest-neighbor thermodynamic models (32, 33, 41) (*SI Appendix*, *Supplementary Note* 1). We also introduce separate parameters for the branch migration activation barriers $\Delta G_{\mathrm{bm}}^{\mathrm{DNA}}$ and $\Delta G_{\mathrm{bm}}^{\mathrm{hybrid}}$ for all-DNA and DNA–RNA systems, respectively.

**Table 3.** Transition rates used in the kinetic model for the default case of RNA>DNA (for other reaction types $\Delta G_{\mathrm{rd}}\left(s,n\right)$ and $\Delta G_{\mathrm{bm}}^{\mathrm{hybrid}}$ are replaced accordingly)

Transition	Forward rate	Backward rate
First toehold base pair	$\frac{c}{c_{0}}k_{\mathrm{bp}}e^{-\Delta G_{\mathrm{assoc}}/k_{B}T}$	$k_{\mathrm{bp}}e^{-\Delta G_{\mathrm{bp}}/k_{B}T}$
Subsequent toehold base pairs	$k_{\mathrm{bp}}$	$k_{\mathrm{bp}}e^{-\Delta G_{\mathrm{bp}}/k_{B}T}$
First displacement step	$k_{\mathrm{bp}}e^{-\left(\Delta G_{\mathrm{bm}}^{\mathrm{hybrid}}+\Delta G_{p}+\frac{1}{2}\Delta G_{\mathrm{rd}}\left(s,1\right)\right)/k_{B}T}$	$k_{\mathrm{bp}}e^{-\left(\Delta G_{\mathrm{bm}}^{\mathrm{hybrid}}-\frac{1}{2}\Delta G_{\mathrm{rd}}\left(s,1\right)\right)/k_{B}T}$
Subsequent displacement steps	$k_{\mathrm{bp}}e^{-\left(\Delta G_{\mathrm{bm}}^{\mathrm{hybrid}}+\frac{1}{2}\Delta G_{\mathrm{rd}}\left(s,n\right)\right)/k_{B}T}$	$k_{\mathrm{bp}}e^{-\left(\Delta G_{\mathrm{bm}}^{\mathrm{hybrid}}-\frac{1}{2}\Delta G_{\mathrm{rd}}\left(s,n\right)\right)/k_{B}T}$
Incumbent unbinding	$\frac{c}{c_{0}}k_{\mathrm{bp}}e^{-\left(-\Delta G_{\mathrm{assoc}}-\Delta G_{\mathrm{bp}}+\frac{1}{2}\Delta G_{\mathrm{rd}}\left(s,N\right)\right)/k_{B}T}$	0

The model is parameterized by a single rate constant ($k_{\mathrm{bp}}$), for base-pair formation during toehold binding, and free energy changes corresponding to the following: the loss of configurational freedom on binding of the incumbent toehold by the invader to form a three-strand complex ($\Delta G_{\mathrm{assoc}}$); formation of an additional DNA–DNA base pair within a duplex ($\Delta G_{\mathrm{bp}}$); an additional penalty for initiating branch migration ($\Delta G_{p}$); an activation barrier to completion of a branch migration step in either direction ($\Delta G_{\mathrm{bm}}^{\mathrm{hybrid}}$); and the stability difference between a DNA–DNA and hybrid base pair ($\Delta G_{\mathrm{rd}}\left(s,n\right)$). c is the strand concentration and $c_{0}=1\;\text{M}$ is the standard reference concentration. The ratio between transition rates between states (except the last) is consistent with detailed balance. At any point along the reaction, with the exception of the initial and final states, the reaction can be terminated early via spontaneous incumbent dissociation which takes place at a rate $k_{\mathrm{bp}}e^{-\sum \Delta G_{\mathrm{bp}}^{*}\left(s,n\right)/k_{B}T}$, where the sum is over all base pairs still formed by the incumbent (including initiation term) and $\Delta G_{\mathrm{bp}}^{*}\left(s,n\right)$ is a modified version of $\Delta G_{\mathrm{bp}}$ intended to capture sequence-dependent effects during dissociation (*SI Appendix*, *Supplementary Note* 5). TMSD is treated as a series of transitions along a 1D Markov chain, and the mean first passage time ⟨t⟩—which can be calculated exactly—is related to the overall reaction rate by $k_{\mathrm{TMSD}}=1/c\left\langle t\right\rangle$. Details of the calculation of ⟨t⟩, adjustments to the model when modeling other reaction types and toehold exchange, as well as the numerical values of the parameters can be found in *SI Appendix*, *Supplementary Note* 5.

### Random Sequences Libraries.

The rate distributions generated using random sequence libraries in Figs. 5 and 6 were robustly reproducible despite covering a small fraction of the sequence space. For a fixed base composition, a string containing all the desired bases was randomly shuffled $10^{5}$ times. For the case of variable base composition sequences of length L, a string containing L bases of each type (total length 4L) was randomly shuffled, and the first L characters of it were saved; again, this procedure was repeated $10^{5}$ times.

## Supplementary Material

Appendix 01 (PDF)

## Data Availability

A *SI Appendix* file is linked to the online version of the article. Simulation files and raw experimental data are available from the repository https://github.com/eryykr/TMSD (57). A Python script implementing the kinetic model is available from the same repository under the filename kinetic_model.py. The standalone oxDNA simulation code, which includes the oxNA model as well as documentation, can be downloaded from https://github.com/lorenzo-rovigatti/oxDNA (58).
