# Supplementary material for: Controlling DNA–RNA strand displacement kinetics with base distribution
Source: Proc Natl Acad Sci U S A. 2025 Jun 6;122(23):e2416988122. doi: 10.1073/pnas.2416988122 (PMC12167940; doi:10.1073/pnas.2416988122)
Supplement: Supplementary file 1 — Appendix 01 (PDF) [file pnas.2416988122.sapp.pdf]

Supplementary Information: "Controlling DNA-RNA strand displacement kinetics with base distribution"

Eryk J. Ratajczyk, Jonathan Bath, Petr Šulc, Jonathan P.K. Doye, Ard A. Louis
and Andrew J. Turberfield

Table of Contents

Supplementary Note 1: Free energy changes during strand displacement **2**

Supplementary Note 2: Full list of sequences and reactions **5**

Supplementary Note 3: Fluorimetry data **6**

Supplementary Note 4: Determination of rate constants with forward flux sampling **8**

Supplementary Note 5: Kinetic model **17**

Supplementary Note 6: Varying toehold length **21**

Supplementary Note 7: Validation of second-order kinetics **22**

Supplementary Note 8: Towards modeling CRISPR-Cas9 **23**

Supplementary Note 9: Screening sequences for secondary structure **25**

Supplementary References **27**

Supplementary Note 1: Free energy changes during strand displacement

Correcting for salt concentration: Throughout this work we use nearest neighbor (NN) parameters from the models of SantaLucia (dsDNA) [7] and Sugimoto (DNA-RNA hybrids) [2]. These models are parameterised at different monovalent salt concentrations of 1 M and 0.1 M respectively. For modelling DNA>DNA reactions, we also employ a set of sequence-dependent coaxial stacking [6] and stacking [4] parameters, which similarly correspond to monovalent salt concentrations of 1 M and 0.1 M respectively.

We apply an empirical salt correction [5] to ΔG values from the models parameterised at 1 M, where $\Delta G^{\circ}_{37}(0.1\,M) = 0.63\,\Delta G^{\circ}_{37}(1\,M) - 1.667$, and ΔG°_{37} is the free energy change of forming an entire duplex in kcal mol^{-1}. When applying this correction to free energy changes over individual displacement steps, as opposed to an entire duplex, we modify it to $\Delta G^{\circ}_{37}(0.1\,M) = 0.63\,\Delta G^{\circ}_{37}(1\,M) - 1.667/N$, where N is the number of bases in the displacement domain.

The empirical salt correction that we use was intended to be applied to the ΔG of a full duplex, and so it is less clear how coaxial stacking parameters should be salt-corrected. We found that simply using $\Delta G^{\circ}_{37}(0.1\,M) = 0.63\,\Delta G^{\circ}_{37}(1\,M)$ worked well, and that including the -1.667 term led to poor predictions at longer strand lengths.

ΔG over an individual displacement step in DNA-RNA systems: Nearest-neighbour models include estimates of the free energy change $\Delta G(XY)$ associated with the formation of every consecutive pair of nucleotides $X, Y \in \{\mathtt{A,T,G,C}\}$ within a duplex. For example, the ΔG°_{37} accompanying the formation of a duplex containing the strand $5'{-}\mathtt{ATGC}{-}3'$ would be calculated as $\Delta G(\mathtt{AT}) + \Delta G(\mathtt{TG}) + \Delta G(\mathtt{GC}) + \Delta G_{init}$, where ΔG_{init} is an initiation penalty. The initiation penalty depends on the model. The dsDNA model has a sequence-independent initiation penalty, as well as a small correction applied if a terminal AT or TA is present within the sequence. For the DNA-RNA hybrid model, the initiation penalty depends on whether the initial base pair is A:U or G:C.

In order to estimate $\Delta G_{rd}(s, n)$, the local free energy changes during branch migration of an RNA>DNA reaction, we compute $\Delta G_{hybrid}(X_n Y_{n+1}) - \Delta G_{DNA}(X_n Y_{n+1})$, where n is the position of a base along the displacement domain. The final free energy change $\Delta G_{rd}(s, N)$, for a displacement domain of length N, is taken to be $\Delta G^{hybrid}_{init} - \Delta G^{DNA}_{init}$, the difference in initiation parameters (the terminal AT correction is very small, and is therefore ignored). For DNA>RNA reactions, we

simply assume that the free energy landscape is inverted, and thus $\Delta G_{dr}(s,n) = -\Delta G_{rd}(s,n)$.

ΔG over an individual displacement step in all-DNA systems: In all-DNA systems, in which base pairs are replaced like-for-like, sequence-dependent effects are more subtle. We consider that a branch migration step changes the position of a nick within the duplex and ignore the contributions to the free energy of the single-stranded overhangs of competing incumbent and invader strands. In the nearest-neighbour model [7], the free energy of forming a nick in a duplex between bases X_nY_{n+1} can be calculated as $\Delta G_{nicked} = \Delta G_{whole} - \Delta G_{DNA}(X_nY_{n+1}) + \Delta G_{cxstck}(X_nY_{n+1}) + \Delta G^{extra}_{init}$, where $\Delta G_{cxstck}(X_nY_{n+1})$ is the coaxial stacking energy between base pairs on either side of the nick which replaces the corresponding NN contribution to the free energy of the intact duplex $\Delta G_{DNA}(X_nY_{n+1})$. ΔG^{extra}_{init} is an additional, sequence-independent, initiation penalty. The energy change between adjacent positions of the nick can thus be written as $\Delta G_{dd}(s,n) = \Delta G_{cxstck}(X_{n+1}Y_{n+2}) - \Delta G_{cxstck}(X_nY_{n+1}) - \Delta G_{DNA}(X_{n+1}Y_{n+2}) + \Delta G_{DNA}(X_nY_{n+1})$. We use coaxial stacking parameters from Peyret and SantaLucia [6].

Bimodal rate distribution in DNA>DNA reactions: The bimodality of the rate distribution for DNA>DNA reactions calculated by the kinetic model (Figure 5(b), main text) is a result of the fixed toehold sequence used in the calculation. The final base of the toehold is `dG`, which has a significant effect on the calculated energy landscape for strand displacement. The rapidly reacting group of sequences in Figure 5(b) contains substrate sequences with one or more `dC` bases at the start of the displacement domain. Our approximation to the energy of the invader bound to the toehold (before invasion of the substrate-incumbent duplex) includes a term corresponding to coaxial stacking across a nick at the beginning of the displacement domain, which in these cases is between strands hybridized to consecutive bases `dGC` of the substrate. This is much weaker than coaxial stacking across the adjacent position of the nick after the first step of strand invasion, opposite substrate sequence `dCX` (where `X` is any base) [6]. This lowers the activation barrier for strand invasion, giving sequences with this motif substantially higher TMSD rates.

ΔG over many displacement steps: In the main text we also introduce the parameter $\Delta G_{RD}(s)$ to quantify the net thermodynamic drive of a reaction, due to the difference in stability between the initial DNA-DNA and final RNA-DNA duplex in the displacement domain. $\Delta G_{RD}(s)$ is thus defined as $\sum_n \Delta G_{rd}(s,n)$, where the sum runs over the length of the displacement domain.

Energy landscapes: For comparison to the energy free energy landscapes in the main text, which were computed using oxNA simulations, we also include landscapes calculated using kinetic model

parameters (including NN parameters) below. Details of how the kinetic model treats all energy changes during TMSD are in Supplementary Note 5.

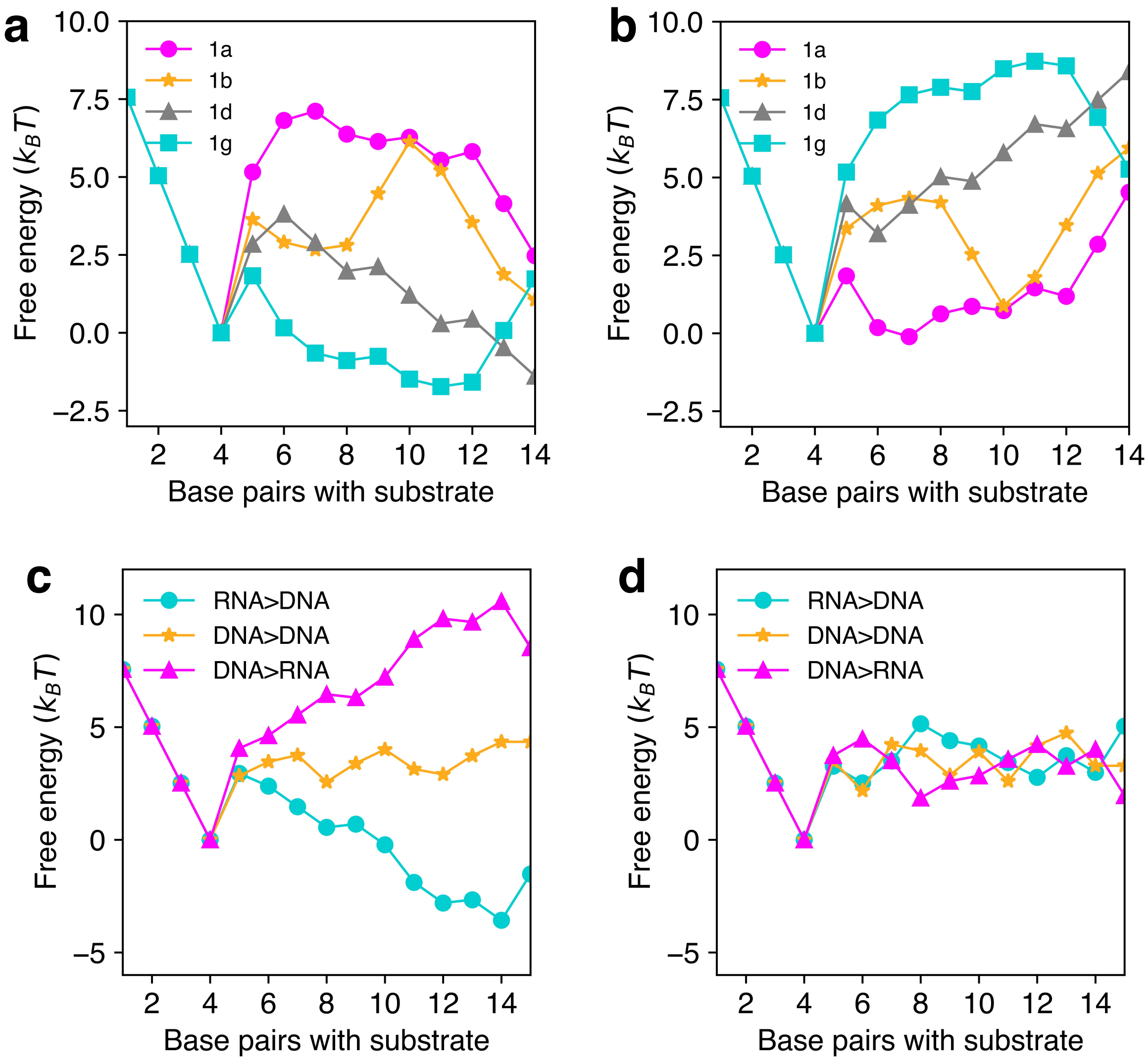

Supplementary Figure 1: Free energy profiles of displacement reactions computed using kinetic model parameters. Selected sequences from Table I for (a) RNA>DNA and (b) DNA>RNA. Reactions from Smith *et al.* [9] for high-purine (c) and low-purine (d) sequences.

Supplementary Note 2: Full list of sequences and reactions

Reaction	Strands (5′ to 3′)
1a, RNA>DNA	dTGTGAAATGTTGCCC (Sub), rGGGCAACAUUUCACA (Inv), 6-FAM–dGGGCAACATTT (Inc)
1a, DNA>RNA	dTGTGAAATGTTGCCC (Sub), dGGGCAACATTTCACA (Inv), 6-FAM–rGGGCAACAUUU (Inc)
1a, DNA>DNA	dTGTGAAATGTTGCCC (Sub), dGGGCAACATTTCACA (Inv), 6-FAM–dGGGCAACATTT (Inc)
1b, RNA>DNA	dTGTGTTGTAAACCCG (Sub), rCGGGUUUACAACACA (Inv), 6-FAM–dCGGGTTTACAA (Inc)
1b, DNA>RNA	dTGTGTTGTAAACCCG (Sub), dCGGGTTTACAACACA (Inv), 6-FAM–rCGGGUUUACAA (Inc)
1b, DNA>DNA	dTGTGTTGTAAACCCG (Sub), dCGGGTTTACAACACA (Inv), 6-FAM–dCGGGTTTACAA (Inc)
1c, RNA>DNA	dTGTGGAAATCGCTCT (Sub), rAGAGCGAUUUCCACA (Inv), 6-FAM–dAGAGCGATTTC (Inc)
1c, DNA>DNA	dTGTGGAAATCGCTCT (Sub), dAGAGCGATTTCCACA (Inv), 6-FAM–dAGAGCGATTTC (Inc)
1d, RNA>DNA	dTGTGGGACTACTACT (Sub), rAGUAGUAGUCCCACA (Inv), 6-FAM–dAGTAGTAGTCC (Inc)
1d, DNA>RNA	dTGTGGGACTACTACT (Sub), dAGTAGTAGTCCCACA (Inv), 6-FAM–rAGUAGUAGUCC (Inc)
1d, DNA>DNA	dTGTGGGACTACTACT (Sub), dAGTAGTAGTCCCACA (Inv), 6-FAM–dAGTAGTAGTCC (Inc)
1e, RNA>DNA	dTGTGTCTGCGAAATC–BHQ1 (Sub), rGAUUUCGCAGACACA (Inv), 6-FAM–dGATTTCGCAGA (Inc)
1e, DNA>DNA	dTGTGTCTGCGAAATC–BHQ1 (Sub), dGATTTCGCAGACACA (Inv), 6-FAM–dGATTTCGCAGA (Inc)
1f, RNA>DNA	dTGTGACTACCATGTG (Sub), rCACAUGGUAGUCACA (Inv), 6-FAM–dCACATGGTAGT (Inc)
1f, DNA>DNA	dTGTGACTACCATGTG (Sub), dCACATGGTAGTCACA (Inv), 6-FAM–dCACATGGTAGT (Inc)
1g, RNA>DNA	dTGTGCCCGTTGTAAA (Sub), rUUUACAACGGGCACA (Inv), 6-FAM–dTTTACAACGGG (Inc)
1g, DNA>DNA	dTGTGCCCGTTGTAAA (Sub), dTTTACAACGGGCACA (Inv), 6-FAM–dTTTACAACGGG (Inc)
1g, DNA>RNA	dTGTGCCCGTTGTAAA (Sub), dTTTACAACGGGCACA (Inv), 6-FAM–rUUUACAACGGG (Inc)
2a, RNA>DNA	dTGTGATGAAAATACT (Sub), rAGUAUUUUCAUCACA (Inv), 6-FAM–dAGTATTTTCAT (Inc)
2a, DNA>DNA	dTGTGATGAAAATACT (Sub), dAGUATTTTCAUCACA (Inv), 6-FAM–dAGTATTTTCAT (Inc)
2b, RNA>DNA	dTGTGTAACCCTGCCG (Sub), rCGGCAGGGUUACACA (Inv), 6-FAM–dCGGCAGGGTTA (Inc)
2b, DNA>DNA	dTGTGTAACCCTGCCG (Sub), dCGGCAGGGTTACACA (Inv), 6-FAM–dCGGCAGGGTTA (Inc)
2c, RNA>DNA	dTGTGCCCTACTTCCC (Sub), rGGGAAGUAGGGCACA (Inv), 6-FAM–dGGGAAGTAGGG (Inc)
2c, DNA>DNA	dTGTGCCCTACTTCCC (Sub), dGGGAAGUAGGGCACA (Inv), 6-FAM–dGGGAAGTAGGG (Inc)
2d, RNA>DNA	dTGTGTCCGTTGAAAA–BHQ1 (Sub), rUUUUCAACGGACACA (Inv), 6-FAM–dTTTTCAACGGA (Inc)
2d, DNA>DNA	dTGTGTCCGTTGAAAA–BHQ1 (Sub), dTTTTCAACGGACACA (Inv), 6-FAM–dTTTTCAACGGA (Inc)
3a, RNA>DNA	dTGTGGAAAAATGCTCGGTCGTCTC–BHQ1 (Sub), rGAGACGACCGAGCAUUUUUCCACA (Inv), 6-FAM–dGAGACGACCGAGCATTTTTC (Inc)
3a, DNA>DNA	dTGTGGAAAAATGCTCGGTCGTCTC–BHQ1 (Sub), dGAGACGACCGAGCATTTTTCCACA (Inv), 6-FAM–dGAGACGACCGAGCATTTTTC (Inc)
3b, RNA>DNA	dTGTGCCCCCAGTGATAATGATGTG–BHQ1 (Sub), rCACAUCAUUAUCACUGGGGGCACA (Inv), 6-FAM–dCACATCATTATCACTGGGGG (Inc)
3b, DNA>DNA	dTGTGCCCCCAGTGATAATGATGTG–BHQ1 (Sub), dCACATCATTATCACTGGGGGCACA (Inv), 6-FAM–dCACATCATTATCACTGGGGG (Inc)
high purine, DNA>RNA	dCATCTCACTACCTACTCGC (Sub), dGTAGGTAGTGAGATG (Inv), rGCGAGUAGGUAGUGA (Inc)
low purine, DNA>RNA	dGTGAGTGAAGTGGAGGTGG (Sub), dCTCCACTTCACTCAC (Inv), rCCACCUCCACUUCAC (Inc)
high purine, DNA>DNA	dCATCTCACTACCTACTCGC (Sub), dGTAGGTAGTGAGATG (Inv), dGCGAGTAGGTAGTGA (Inc)
low purine, DNA>DNA	dGTGAGTGAAGTGGAGGTGG (Sub), dCTCCACTTCACTCAC (Inv), dCCACCTCCACTTCAC (Inc)
high purine, RNA>DNA	dCATCTCACTACCTACTCGC (Sub), rGUAGGUAGUGAGAUG (Inv), dGCGAGTAGGTAGTGA (Inc)
low purine, RNA>DNA	dGTGAGTGAAGTGGAGGTGG (Sub), rCUCCACUUCACUCAC (Inv), dCCACCTCCACTTCAC (Inc)

Supplementary Table 1: Full set of sequences used for each reaction characterized experimentally in this work. We also include sequences from Smith *et al.* [9], which were simulated. 6-FAM indicates the fluorescent dye modification and BHQ1 indicates a black hole quencher.

Supplementary Note 3: Fluorimetry data

Generally, the fit of fluorescence intensity to the expected second-order curve was excellent. Every reaction was repeated three times. The full dataset can be found in the GitHub repository referred to in the main text.

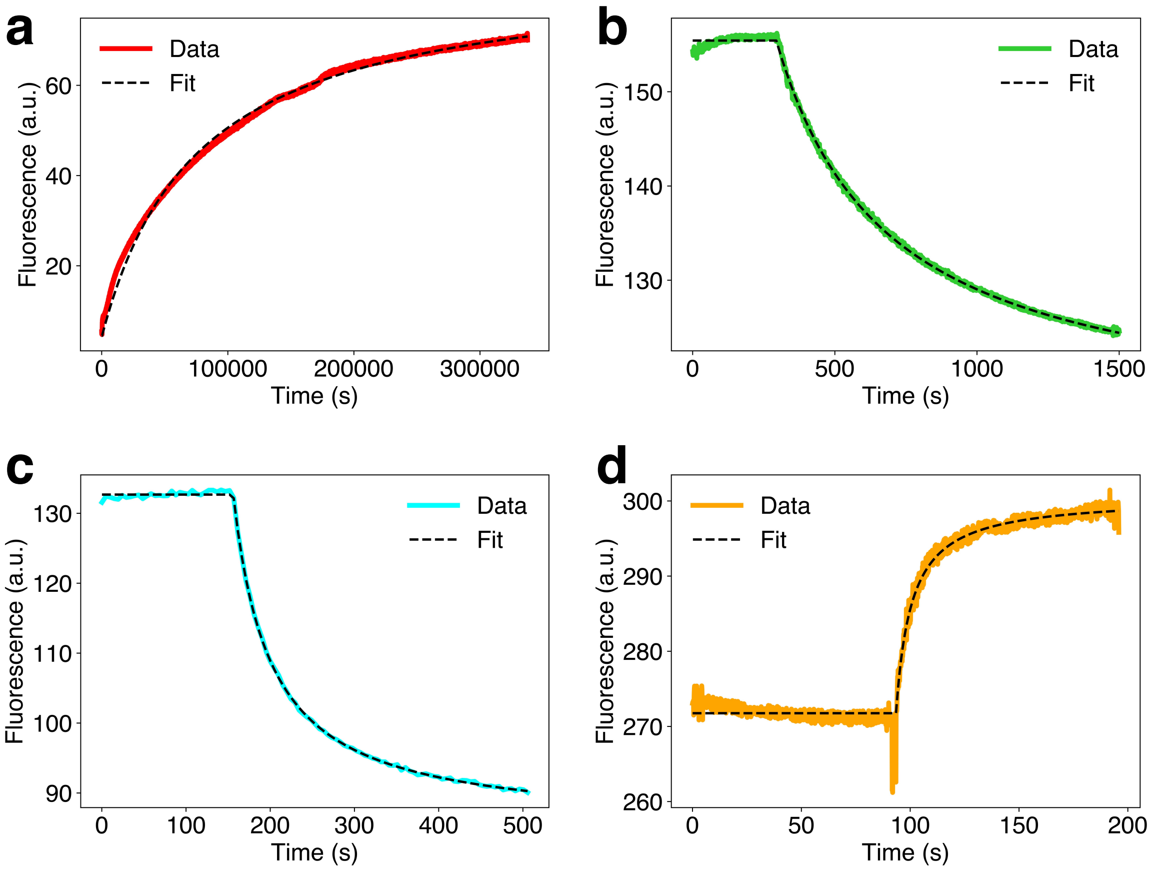

Supplementary Figure 2: Examples of experimental fluorescence traces with second-order fits, for (a) sequence 3a, RNA>DNA, (b) sequence 1a, RNA>DNA, (c) sequence 2c, RNA>DNA and (d) sequence 1f, DNA>DNA.

Reaction	Rate Constants ($M^{-1}s^{-1}$)
1a, RNA>DNA	2.53×10^3, 2.83×10^3, 2.58×10^3
1a, DNA>RNA	2.85×10^5, 2.55×10^5, 2.58×10^5
1a, DNA>DNA	6.14×10^4, 6.68×10^4, 5.56×10^4
1b, RNA>DNA	2.98×10^3, 4.73×10^3, 5.82×10^3
1b, DNA>RNA	9.55×10^3, 1.07×10^4, 1.12×10^4
1b, DNA>DNA	5.46×10^3, 5.10×10^3, 6.68×10^3
1c, RNA>DNA	5.88×10^3, 7.20×10^3, 6.53×10^3
1c, DNA>DNA	3.48×10^4, 3.54×10^4, 3.54×10^4
1d, RNA>DNA	7.17×10^4, 5.75×10^4, 8.30×10^4
1d, DNA>RNA	2.03×10^4, 2.27×10^4, 2.65×10^4
1d, DNA>DNA	5.85×10^4, 5.80×10^4, 6.19×10^4
1e, RNA>DNA	2.07×10^5, 1.86×10^5, 1.99×10^5
1e, DNA>DNA	3.20×10^4, 3.35×10^4, 3.30×10^4
1f, RNA>DNA	2.86×10^5, 2.57×10^5, 2.43×10^5
1f, DNA>DNA	1.39×10^5, 1.74×10^5, 1.05×10^5
1g, RNA>DNA	8.50×10^5, 8.20×10^5, 1.13×10^6
1g, DNA>RNA	1.92×10^4, 1.92×10^4, 1.98×10^4
1g, DNA>DNA	1.32×10^5, 1.50×10^5, 1.31×10^5
2a, RNA>DNA	1.04×10^4, 6.79×10^3, 8.16×10^3
2a, DNA>DNA	2.41×10^4, 2.22×10^4, 2.20×10^4
2b, DNA>DNA	6.32×10^4, 5.82×10^4, 6.92×10^4
2b, RNA>DNA	2.03×10^5, 1.88×10^5, 2.01×10^5
2c, RNA>DNA	4.47×10^5, 4.65×10^5, 4.55×10^5
2c, DNA>DNA	2.38×10^5, 2.55×10^5, 2.37×10^5
2d, RNA>DNA	1.18×10^6, 1.17×10^6, 1.40×10^6
2d, DNA>DNA	1.85×10^5, 2.00×10^5, 2.00×10^5
3a, RNA>DNA	2.63×10^1, 2.39×10^1, 4.26×10^1
3b, DNA>DNA	1.01×10^3, 1.02×10^3, 1.05×10^3
3a, RNA>DNA	5.02×10^5, 5.33×10^5, 5.20×10^5
3b, DNA>DNA	8.74×10^4, 8.22×10^4, 9.98×10^4

Supplementary Table 2: Complete list of fitted rate constants of each reaction studied, from three replicate experiments. Mean rate constants are quoted in the main text.

Supplementary Note 4: Determination of rate constants with forward flux sampling

Simulation protocol: Below we provide additional details of forward flux sampling simulations [1]. The partitioning scheme of the reaction coordinate in terms of an order parameter Q can be found in Supplementary Table 3. Interface λ_y^x denotes reaching $Q = x$ from a state at $Q = y$. Simulations were run in parallel on 20 CPUs. To estimate initial flux (interface λ_{-1}^0), the mean time taken for the system to reach $Q = 0$ was measured and, for each success, the configuration was saved. For all subsequent interfaces λ_n^{n+1} new simulations are launched, using the previously saved successes as starting configurations. The system can either reach $Q = n + 1$ or return to $Q = -2$, and we estimate the probability of a successful forward crossing. For interfaces in which the limiting process is diffusion or hybridization, we launch new simulations until at least 1000 successful crossings are recorded. For interfaces which require strand displacement, which is much slower, at least 100 successful crossings are required. In some cases fewer than 100 successes were recorded if the interface happened to be especially slow.

Detailed forward flux sampling data for all of the newly designed reactions presented in the main text are found in Supplementary Tables 4–18. We have only included data from simulations of RNA>DNA and DNA>RNA reactions. Since the oxDNA model is unable to correctly capture the sequence-dependence among DNA>DNA reactions, additional details about these simulations are unlikely to be of any use. For simulations of toehold exchange reactions taken from Smith *et al.* [9], see Supplementary Tables 19–24. For each reaction, the effective rate constant is taken to be the product of all the values in the final column.

Limitations of oxDNA2: The oxDNA2 model is unable to reproduce experimentally observed DNA>DNA sequence-dependent kinetics (main text, Table I). This highlights a limitation of the oxDNA2 model: although stacking interactions are sequence-dependent, coaxial stacking is sequence-independent. For DNA>DNA, the kinetic model uses sequence-dependent coaxial stacking parameters [6]; this was found to be the only parameter set that could explain experimental observations.

Limitations of oxNA: In Table II of the main text, our simulations underestimate the rate of the low purine RNA>DNA reaction by over two orders of magnitude. Inspection of the FFS data (Supplementary Table 24) reveals a single interface which is very difficult to cross, and is associated with replacing a pair of `dT:dA` base pairs by `rU:dA`. This suggests that oxNA may be underestimating

the strength of `rU:dA` base pairs. oxNA was parameterised in way which ensures compatibility with oxDNA and oxRNA, namely, intra-strand interactions were inherited from these models, and only the DNA-RNA hydrogen bonding interaction was parameterised from scratch. A future version of oxNA, in which all interactions are reparameterised, is likely to reproduce experimental observations even better.

**Order parameter**	**Criterion**
$Q = -2$	$d > 4$
$Q = -1$	$d \leq 4$
$Q = 0$	$d \leq 1$
$Q = 1$	$N \geq 1$
$Q = 2$	$N \geq 4$
$Q = 3$	$N \geq 7$
$Q = 4$	$N \geq 12$
$Q = 5$	$N \geq 15$
$Q = 6^{\dagger}$	$d_{TX} \geq 4$

Supplementary Table 3: Order parameters used in forward flux sampling. We define d as the shortest out of all pairwise distances between complementary nucleotides in the toehold and in the invader's toehold binding domain. d_{TX} is defined analogously, but for the distal toehold, applicable to toehold exchange reactions only. N is the number of base pairs between the invader and substrate. Two nucleotides are considered base paired if their hydrogen bonding energy is less than -0.1 energy units (1 energy unit is equal to the thermal energy k_BT at $T = 3000\,K$). †The order parameter $Q = 6$ was only used in simulations of toehold exchange.

**Interface**	**Crossings**	**Mean time (dt)**	**Flux (dt^{-1})**
λ_{-1}^{0}	1001	823727	1.21399×10^{-6}
**Interface**	**Crossings**	**Attempts**	**Probability**
λ_{0}^{1}	1000	80228	0.0124645
λ_{1}^{2}	1009	6410	0.15741
λ_{2}^{3}	117	160	0.73125
λ_{3}^{4}	40	4506	0.00887705
λ_{4}^{5}	101	246	0.410569

Supplementary Table 4: Initial flux, and forward crossing probabilities of all interfaces, for sequence 1a, RNA>DNA.

**Interface**	**Crossings**	**Mean time (dt)**	**Flux (dt^{-1})**
λ^0_{-1}	1001	1213360	8.2416×10^{-7}

**Interface**	**Crossings**	**Attempts**	**Probability**
λ^1_0	1007	153327	0.00656766
λ^2_1	1003	5084	0.197286
λ^3_2	107	233	0.459227
λ^4_3	103	1194	0.0862647
λ^5_4	103	216	0.476852

Supplementary Table 5: Initial flux, and forward crossing probabilities of all interfaces, for sequence 1b, RNA>DNA.

**Interface**	**Crossings**	**Mean time (dt)**	**Flux (dt^{-1})**
λ^0_{-1}	1001	993515	1.00653×10^{-6}

**Interface**	**Crossings**	**Attempts**	**Probability**
λ^1_0	1001	133357	0.00750617
λ^2_1	1013	4561	0.2221
λ^3_2	116	131	0.885496
λ^4_3	30	5368	0.00558867
λ^5_4	115	119	0.966387

Supplementary Table 6: Initial flux, and forward crossing probabilities of all interfaces, for sequence 1c, RNA>DNA.

**Interface**	**Crossings**	**Mean time (dt)**	**Flux (dt^{-1})**
λ^0_{-1}	1001	1003520	9.96491×10^{-7}

**Interface**	**Crossings**	**Attempts**	**Probability**
λ^1_0	1002	94444	0.0106095
λ^2_1	1010	9956	0.101446
λ^3_2	109	177	0.615819
λ^4_3	109	548	0.198905
λ^5_4	116	119	0.97479

Supplementary Table 7: Initial flux, and forward crossing probabilities of all interfaces, for sequence 1d, RNA>DNA.

**Interface**	**Crossings**	**Mean time (dt)**	**Flux (dt^{-1})**
λ_{-1}^0	1001	935979	1.0684×10^{-6}

**Interface**	**Crossings**	**Attempts**	**Probability**
λ_0^1	1002	129827	0.00771796
λ_1^2	1002	5057	0.198141
λ_2^3	115	134	0.858209
λ_3^4	105	710	0.147887
λ_4^5	117	119	0.983193

Supplementary Table 8: Initial flux, and forward crossing probabilities of all interfaces, for sequence 1e, RNA>DNA.

**Interface**	**Crossings**	**Mean time (dt)**	**Flux (dt^{-1})**
λ_{-1}^0	1000	1004800	9.9522×10^{-7}

**Interface**	**Crossings**	**Attempts**	**Probability**
λ_0^1	1000	177234	0.00564226
λ_1^2	1008	6070	0.166063
λ_2^3	111	175	0.634286
λ_3^4	106	558	0.189964
λ_4^5	114	122	0.934426

Supplementary Table 9: Initial flux, and forward crossing probabilities of all interfaces, for sequence 1f, RNA>DNA.

**Interface**	**Crossings**	**Mean time (dt)**	**Flux (dt^{-1})**
λ_{-1}^0	1001	998767	1.00123×10^{-6}

**Interface**	**Crossings**	**Attempts**	**Probability**
λ_0^1	1000	121033	0.00826221
λ_1^2	1013	5022	0.201712
λ_2^3	116	145	0.8
λ_3^4	110	180	0.611111
λ_4^5	118	119	0.991597

Supplementary Table 10: Initial flux, and forward crossing probabilities of all interfaces, for sequence 1g, RNA>DNA.

**Interface**	**Crossings**	**Mean time (dt)**	**Flux ($\mathbf{dt^{-1}}$)**
λ_{-1}^{0}	1001	909103	1.09999×10^{-6}

**Interface**	**Crossings**	**Attempts**	**Probability**
λ_0^1	1003	137835	0.00727682
λ_1^2	1006	4195	0.239809
λ_2^3	106	293	0.361775
λ_3^4	103	995	0.103518
λ_4^5	102	124	0.822581

Supplementary Table 11: Initial flux, and forward crossing probabilities of all interfaces, for sequence 2a, RNA>DNA.

**Interface**	**Crossings**	**Mean time (dt)**	**Flux ($\mathbf{dt^{-1}}$)**
λ_{-1}^{0}	1003	948735	1.05404×10^{-6}

**Interface**	**Crossings**	**Attempts**	**Probability**
λ_0^1	1005	157321	0.00638821
λ_1^2	1001	3551	0.281892
λ_2^3	113	140	0.807143
λ_3^4	48	1357	0.0353721
λ_4^5	111	136	0.816176

Supplementary Table 12: Initial flux, and forward crossing probabilities of all interfaces, for sequence 2b, RNA>DNA.

**Interface**	**Crossings**	**Mean time (dt)**	**Flux ($\mathbf{dt^{-1}}$)**
λ_{-1}^{0}	1001	975753	1.02485×10^{-6}

**Interface**	**Crossings**	**Attempts**	**Probability**
λ_0^1	1001	204045	0.00490578
λ_1^2	1015	2801	0.362371
λ_2^3	115	166	0.692771
λ_3^4	117	139	0.841727
λ_4^5	119	121	0.983471

Supplementary Table 13: Initial flux, and forward crossing probabilities of all interfaces, for sequence 2c, RNA>DNA.

**Interface**	**Crossings**	**Mean time (dt)**	**Flux (dt^{-1})**
λ_{-1}^{0}	1000	976153	1.02443×10^{-6}

**Interface**	**Crossings**	**Attempts**	**Probability**
λ_{0}^{1}	1002	145699	0.00687719
λ_{1}^{2}	1004	4453	0.225466
λ_{2}^{3}	113	170	0.664706
λ_{3}^{4}	113	232	0.487069
λ_{4}^{5}	119	119	1

Supplementary Table 14: Initial flux, and forward crossing probabilities of all interfaces, for sequence 2d, RNA>DNA.

**Interface**	**Crossings**	**Mean time (dt)**	**Flux (dt^{-1})**
λ_{-1}^{0}	1000	1416690	7.05873×10^{-7}

**Interface**	**Crossings**	**Attempts**	**Probability**
λ_{0}^{1}	1001	475371	0.00210572
λ_{1}^{2}	1000	7038	0.142086
λ_{2}^{3}	117	131	0.89313
λ_{3}^{4}	112	144	0.777778
λ_{4}^{5}	117	125	0.936

Supplementary Table 15: Initial flux, and forward crossing probabilities of all interfaces, for sequence 1b, DNA>RNA.

**Interface**	**Crossings**	**Mean time (dt)**	**Flux (dt^{-1})**
λ_{-1}^{0}	1000	1243360	8.04271×10^{-7}

**Interface**	**Crossings**	**Attempts**	**Probability**
λ_{0}^{1}	1000	201376	0.00496584
λ_{1}^{2}	1008	14239	0.0707915
λ_{2}^{3}	106	362	0.292818
λ_{3}^{4}	103	520	0.198077
λ_{4}^{5}	117	118	0.983193

Supplementary Table 16: Initial flux, and forward crossing probabilities of all interfaces, for sequence 1g, DNA>RNA.

**Interface**	**Crossings**	**Mean time (dt)**	**Flux (dt^{-1})**
λ_{-1}^{0}	1001	1289650	7.75404×10^{-7}

**Interface**	**Crossings**	**Attempts**	**Probability**
λ_0^1	1000	188950	0.00529241
λ_1^2	1011	8692	0.116314
λ_2^3	115	147	0.782313
λ_3^4	114	202	0.564356
λ_4^5	112	122	0.918033

Supplementary Table 17: Initial flux, and forward crossing probabilities of all interfaces, for sequence 1d, DNA>RNA.

**Interface**	**Crossings**	**Mean time (dt)**	**Flux (dt^{-1})**
λ_{-1}^{0}	1001	1254110	7.97377×10^{-7}

**Interface**	**Crossings**	**Attempts**	**Probability**
λ_0^1	1000	124554	0.00802865
λ_1^2	1009	3267	0.308846
λ_2^3	119	121	0.983471
λ_3^4	118	125	0.944
λ_4^5	118	127	0.929134

Supplementary Table 18: Initial flux, and forward crossing probabilities of all interfaces, for sequence 1a, DNA>RNA.

**Interface**	**Crossings**	**Mean time (dt)**	**Flux (dt^{-1})**
λ_{-1}^{0}	1000	8035860	1.24442×10^{-7}

**Interface**	**Crossings**	**Attempts**	**Probability**
λ_0^1	1000	96407	0.0103727
λ_1^2	1017	8974	0.113327
λ_2^3	108	316	0.341772
λ_3^4	108	340	0.317647
λ_4^5	103	350	0.294286
λ_5^6	30	404	0.0742574

Supplementary Table 19: Initial flux, and forward crossing probabilities of all interfaces, for high purine DNA>RNA.

Interface	Crossings	Mean time (dt)	Flux (dt^{-1})
λ_{-1}^{0}	1001	7955630	1.25697×10^{-7}

Interface	Crossings	Attempts	Probability
λ_{0}^{1}	1002	130690	0.007667
λ_{1}^{2}	1001	4155	0.240915
λ_{2}^{3}	118	154	0.766234
λ_{3}^{4}	120	143	0.839161
λ_{4}^{5}	121	125	0.968
λ_{5}^{6}	123	127	0.968504

Supplementary Table 20: Initial flux, and forward crossing probabilities of all interfaces, for low purine DNA>RNA.

Interface	Crossings	Mean time (dt)	Flux (dt^{-1})
λ_{-1}^{0}	1001	7739150	1.29213×10^{-7}

Interface	Crossings	Attempts	Probability
λ_{0}^{1}	1001	117702	0.00850453
λ_{1}^{2}	1015	7841	0.129448
λ_{2}^{3}	107	274	0.390511
λ_{3}^{4}	106	313	0.338658
λ_{4}^{5}	111	162	0.685185
λ_{5}^{6}	105	221	0.475113

Supplementary Table 21: Initial flux, and forward crossing probabilities of all interfaces, for high purine DNA>DNA.

Interface	Crossings	Mean time (dt)	Flux (dt^{-1})
λ_{-1}^{0}	1001	7924070	1.26198×10^{-7}

Interface	Crossings	Attempts	Probability
λ_{0}^{1}	1002	117725	0.00851136
λ_{1}^{2}	1011	3843	0.263076
λ_{2}^{3}	113	248	0.455645
λ_{3}^{4}	105	326	0.322086
λ_{4}^{5}	106	196	0.540816
λ_{5}^{6}	109	233	0.467811

Supplementary Table 22: Initial flux, and forward crossing probabilities of all interfaces, for low purine DNA>DNA.

**Interface**	**Crossings**	**Mean time (dt)**	**Flux ($\mathbf{dt^{-1}}$)**
λ_{-1}^0	1000	7673070	1.30326×10^{-7}

**Interface**	**Crossings**	**Attempts**	**Probability**
λ_0^1	1000	174838	0.00571958
λ_1^2	1020	2541	0.401417
λ_2^3	120	131	0.916031
λ_3^4	116	207	0.560386
λ_4^5	113	150	0.753333
λ_5^6	121	141	0.858156

Supplementary Table 23: Initial flux, and forward crossing probabilities of all interfaces, for high purine RNA>DNA.

**Interface**	**Crossings**	**Mean time (dt)**	**Flux ($\mathbf{dt^{-1}}$)**
λ_{-1}^0	1000	7036900	1.42108×10^{-7}

**Interface**	**Crossings**	**Attempts**	**Probability**
λ_0^1	1002	166665	0.00601206
λ_1^2	1002	3044	0.329172
λ_2^3	106	391	0.2711
λ_3^4	101	2424	0.0416667
λ_4^5	101	897	0.112598
λ_5^6	102	1073	0.0950606

Supplementary Table 24: Initial flux, and forward crossing probabilities of all interfaces, for low purine RNA>DNA.

Supplementary Note 5: Kinetic model

The model is parameterized by a rate constant k_{bp}, which fixes the absolute timescale of transitions, and a series of free energy changes that set the relative rates of transitions between adjacent states: the energy to form a base pair with the toehold ΔG_{bp}, the energy required to bring a strand out of solution (accompanied by a significant entropy loss) as it binds to the toehold ΔG_{assoc}, and an energy required to initiate branch migration ΔG_p. For all of the above parameters we use the same values as Smith *et al.* [9]. Newly introduced parameters are discussed below.

A given branch migration step is assumed to have an activation barrier ΔG_{bm}^{DNA} or ΔG_{bm}^{hybrid}, corresponding to all-DNA and DNA-RNA strand displacement respectively, whose values were chosen so that the model best reproduces experimentally observed reaction rates. It was necessary to use two separate parameters for all-DNA and DNA-RNA systems in order to adequately capture the absolute rates of all reaction types using a single model. These activation barriers are expected to be larger than the energy to melt a base pair ΔG_{bp}, in order to account for other factors contributing to the relative slowness of branch migration, such as steric effects. These activation barriers are also not necessarily the same for all-DNA and DNA-RNA strand displacement, given the different structures and flexibilities of DNA and RNA strands. This sequence-independent barrier is applied to branch migration steps in both directions, and sequence-dependent energy changes are captured by one of $\Delta G_{rd}(s, n)$, $\Delta G_{dr}(s, n)$ and $\Delta G_{dd}(s, n)$. These parameters, dependent on sequence s and branch migration step n, quantify the net free energy change of a single forward branch migration step. For RNA>DNA we use $\Delta G_{rd}(s, n)$, for DNA>RNA we assume $\Delta G_{dr}(s, n) = -\Delta G_{rd}(s, n)$, and for DNA>DNA we use $\Delta G_{dd}(s, n)$. Details of how these are computed are provided in Supplementary Note 1, and their exact values can be found in the Python script implementing the kinetic model (see main text).

Model parameters: $k_{bp} = 6.4\times10^7\,\mathrm{s}^{-1}$, $\Delta G_{bp} = 2.52$, $\Delta G_{assoc} = 2.5$, $\Delta G_{bm}^{hybrid} = 9.48$, $\Delta G_{bm}^{DNA} = 10.3$ and $\Delta G_p = 3.5$ (in units of k_BT). We also set $c = 1\,\mu\mathrm{M}$ and $c_0 = 1\,\mathrm{M}$.

The default average-sequence base pairing free energy used in the kinetic model, $\Delta G_{bp} = 2.52$, is significantly larger in magnitude than nearest-neighbour model estimates (around $1.5\,k_BT$ for dsDNA). Thus, directly replacing ΔG_{bp} with NN parameters for base pairing free energies would lead to overestimation of dissociation rates. We define an energy offset ΔG_0 such that $\Delta G_{bp} = \Delta G_0 + \Delta G_{NN}^{avg}$, where ΔG_{NN}^{avg} is an average-sequence estimate of the base pair formation free energy

(ΔG_{NN}^{avg} is $1.49\, k_B T$ for dsDNA and $2.01\, k_B T$ for hybrids). To calculate a sequence-dependent activation energy for spontaneous dissociation of the last m bases of the incumbent, containing base sequence s, we use a modified nearest-neighbour free energy $\Delta G_{bp}^{*}(s,n) = \Delta G_0 + \Delta G_{NN}(s,n)$. The activation energy for dissociation of the incumbent is taken as $\sum \Delta G_{NN}(s,n)$, where the sum runs over the remaining base pairs formed by the incumbent. We include the duplex initiation penalty. We find that including sequence-dependent dissociation improves the model's fit to experimental reaction rates.

Strand association/dissociation is also concentration-dependent, captured by parameters c, the strand concentration, and c_0, a reference concentration.

Determining transition rates from energy changes: At equilibrium, the rates of forward and backward transitions between adjacent states n and $n+1$ are constrained by detailed balance, such that

$$\frac{k_n^+}{k_{n+1}^-} = \exp\left(-\frac{G_{n+1} - G_n}{k_B T}\right), \tag{1}$$

where G_{n+1} and G_n are the free energies of states $n+1$ and n respectively.

Modeling branch migration: Given that the focus of the work is on the effect of displacement domain sequence on reaction kinetics, particular attention was paid to constructing a plausible model of branch migration. Branch migration has a relatively high activation barrier; sequence effects are expected to modulate the height of this barrier. However, how the activation energy landscape should depend on the different free energies of the competing base pairs is not clear. Previous oxDNA simulation work [10] has suggested two possible mechanisms of the branch migration process. In the discussion below we use example of RNA>DNA.

In one mechanism, the incumbent base pair first fully breaks, and can then be replaced by an invading base pair. This was found to be the more common mechanism at later stages of branch migration. In this scenario, one could reasonably assume that the forward rate depends entirely on the strength of the base pair being replaced, and the backward rate on the invading base pair. One could thus assign rates as follows: $k_{bm}^{forward} = k_{bm} e^{-\Delta G_{DNA}}$ and $k_{bm}^{backward} = k_{bm} e^{-\Delta G_{hybrid}}$, where k_{bm} is a scale factor that sets the time scale for branch migration.

The second mechanism, found to be most common at the early stages of branch migration, involves

an invading base pair displacing the incumbent one more gradually, without it fully breaking first—the invading and incumbent bases compete over binding to the substrate until one is displaced. In modeling this mechanism, one could assume that *both* the forward and backward steps depend on the difference in strength between incumbent and invading base pairs ΔG_{rd}.

While the simulation work discussed above may provide some insight, the exact mechanism of branch migration is not known, and behaviours observed in oxDNA simulations may be artefacts of coarse-graining. We trialed a number of different models, and found that the following worked best: $k_{bm}^{forward} = k_{bm}e^{\Delta G_{rd}/2}$ and $k_{bm}^{backward} = k_{bm}e^{-\Delta G_{rd}/2}$. This satisfies detailed balance, as well as the plausible assumption that both forward and backward rates should be affected by the relative stabilities of the outgoing and incoming base pairs. Moreover, it produces the best fit to experimental among all of the variations explored.

Transition rates: Exact expressions for transition rates are given in Supplementary Table 25, which can also be found in the main text. The final few transitions in the model are different depending on whether a second toehold is present, as indicated in the table.

Calculating the rate of a displacement reaction: The strand displacement reaction is represented as a 1D Markov chain—an invading strand starts unbound in solution and forms an increasing number of base pairs with the substrate. The final displacement step is accompanied by irreversible incumbent dissociation. We also allow the incumbent to spontaneously dissociate at each state.

The system is defined by $N+1$ positions along a chain labeled as $\{0, 1, ..., N\}$, with a reflecting boundary at 0 and an absorbing boundary at N. Adjacent state probabilities are related by

$$p_n = \frac{j_n}{k_n^+} + \frac{k_{n+1}^-}{k_n^+} p_{n+1} \tag{2}$$

and in steady-state it is true that

$$j_n = j_{n+1} + k_{n+1}^{off} p_{n+1}, \tag{3}$$

where j_n is the flux between positions n and $n+1$, k_n^+ is the (forward) rate from n to $n+1$, k_{n+1}^- is the (backward) rate from $n+1$ to n, and k_n^{off} is rate between position n and position 0 (corresponding to spontaneous dissociation). It can be shown that the mean first passage time is

$$\langle t \rangle = \frac{j_{N-1}}{j_0} \sum_{n=0}^{N} \frac{p_n}{j_{N-1}}, \tag{4}$$

Transition	Forward Rate	Backward Rate
First toehold base pair	$\frac{c}{c_0}k_{bp}e^{-\Delta G_{assoc}/k_BT}$	$k_{bp}e^{-\Delta G_{bp}/k_BT}$
Subsequent toehold base pairs	k_{bp}	$k_{bp}e^{-\Delta G_{bp}/k_BT}$
First displacement step	$k_{bp}e^{-(\Delta G_{bm}^{hybrid}+\Delta G_p+\frac{1}{2}\Delta G_{rd}(s,1))/k_BT}$	$k_{bp}e^{-(\Delta G_{bm}^{hybrid}-\frac{1}{2}\Delta G_{rd}(s,1))/k_BT}$
Subsequent displacement steps	$k_{bp}e^{-(\Delta G_{bm}^{hybrid}+\frac{1}{2}\Delta G_{rd}(s,n))/k_BT}$	$k_{bp}e^{-(\Delta G_{bm}^{hybrid}-\frac{1}{2}\Delta G_{rd}(s,n))/k_BT}$
Incumbent unbinding (if TMSD)	$\frac{c}{c_0}k_{bp}e^{-(-\Delta G_{assoc}-\Delta G_{bp}+\frac{1}{2}\Delta G_{rd}(s,N))/k_BT}$	0
Final displacement step (if TX)	$k_{bp}e^{-(-\Delta G_p+\frac{1}{2}\Delta G_{rd}(s,N)+\Delta G_{bm}^{hybrid})/k_BT}$	$k_{bp}e^{-\Delta G_{bm}/k_BT}$
Breaking toehold base pairs (if TX)	$k_{bp}e^{-\Delta G_{bp}/k_BT}$	k_{bp}
Incumbent unbinding (if TX)	$\frac{c}{c_0}k_{bp}e^{-(-\Delta G_{assoc}+\Delta G_{bp}-\Delta G_{bp})/k_BT}$	0

Supplementary Table 25: Transition rates used in the kinetic model for the default case of RNA>DNA (for other reaction types $\Delta G_{rd}(s,n)$ and ΔG_{bm}^{hybrid} would be replaced accordingly). The first four rows of the table are common to all reaction types studied (toehold exchange is abbreviated as TX). The model is parameterized by base pair formation rate k_{bp} (which is not sequence-dependent) and free energy changes corresponding to: base pairing (ΔG_{bp}), binding of the incumbent toehold by the invader to form a three-strand complex (ΔG_{assoc}), completion of a branch migration step in either direction (ΔG_{bm}^{hybrid}), an additional penalty for initiating branch migration (ΔG_p), and the stability difference between dsDNA and hybrids ($\Delta G_{rd}(s,n)$). c is the strand concentration, and c_0 is a reference concentration. The relative transition rates between states with a given free energy change are set by detailed balance. At any point along the reaction, with the exception of the initial and final state, as well as during the unbinding of the second toehold in the case of toehold exchange, the reaction can be terminated early via spontaneous incumbent dissociation which takes place at the rate $k_{bp}e^{-\sum \Delta G_{bp}^*(s)/k_BT}$, where the sum is over all base pairs still formed by the incumbent (including initiation term), and $\Delta G_{bp}^*(s)$ is a modified version of ΔG_{bp} intended to capture sequence-dependent effects during dissociation. TMSD is treated as a series of transitions along a 1D Markov chain, and the mean first passage time $\langle t \rangle$—which can be calculated exactly—is related to the overall reaction rate by $k_{TMSD} = 1/c\langle t \rangle$.

and the overall displacement rate is simply $k_{TMSD} = 1/c\langle t \rangle$

Supplementary Note 6: Varying toehold length

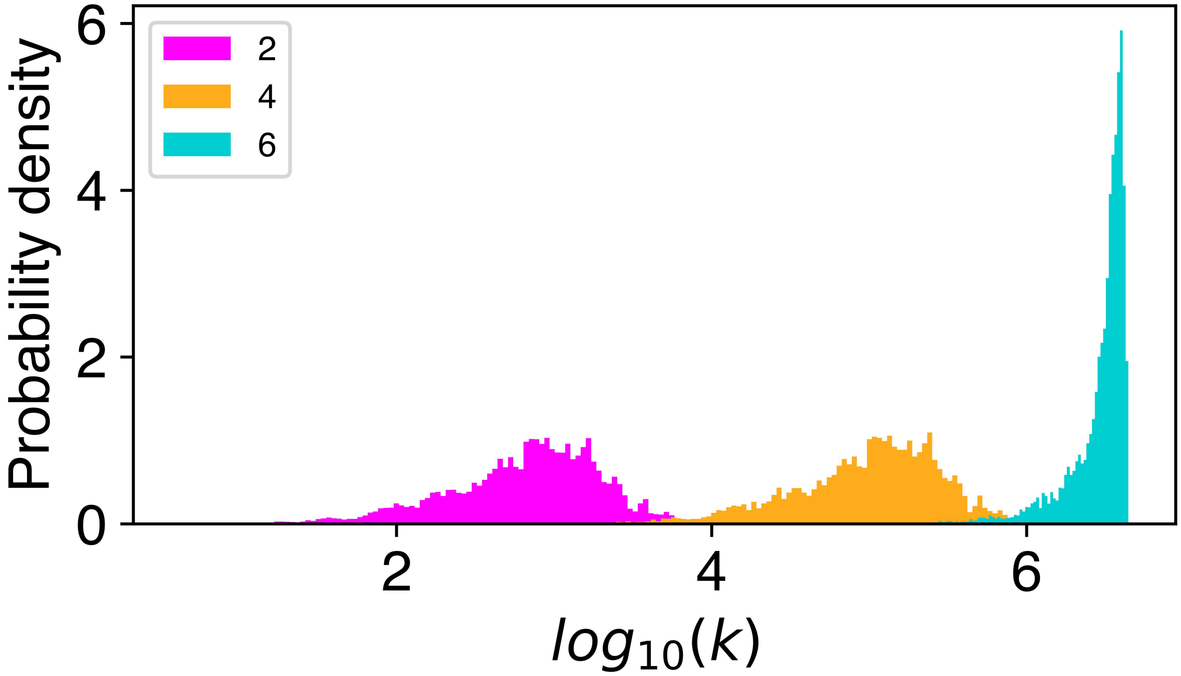

Supplementary Figure 3: Effect of toehold length on reaction kinetics. Base distributions of a random library of 10^5 sequences of fixed base composition of length 11.

Supplementary Note 7: Validation of second-order kinetics

The assumption of second-order kinetics was validated by repeating the reaction with sequence 2b, RNA>DNA, at three additional concentrations. The fitted, concentration-unadjusted rate constant scales with concentration as expected.

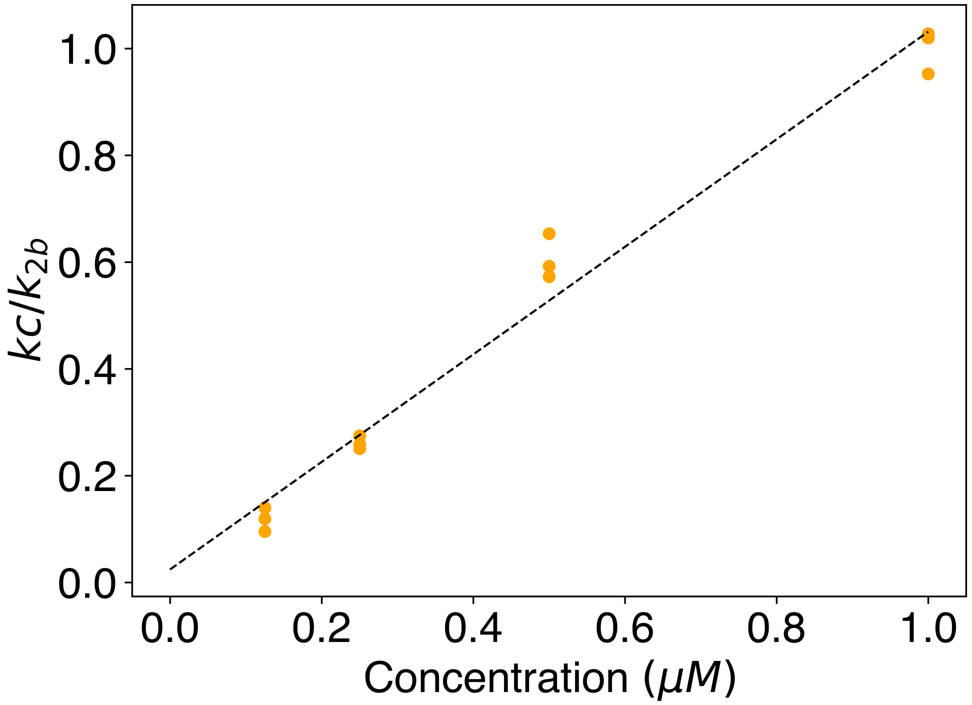

Supplementary Figure 4: Apparent rate constant (not adjusted for concentration) of the reaction with 2b, RNA>DNA, as a function of concentration, and a least squares fit ($y = 1.00x + 0.02$).

Supplementary Note 8: Towards modeling CRISPR-Cas9

Sequence-independence of $\Delta G_{Cas9}(n)$: The distinguishing feature of the CRISPR-Cas9 free energy landscape is a metastable state/local minimum at around 10bp, which can be seen in Figure 6(a) in the main text. This metastable state has been observed experimentally for different target sequences [8, 3]. In general, interactions between Cas9 and DNA/RNA are unlikely to be strictly sequence-independent, but those that have the strongest effect on the energy landscape can be approximated as sequence-independent. This is also the reason that we model these interactions as position-dependent: there is a metastable state at around 10bp, which is present regardless of the sequence, and in order to capture the underlying shape of the Cas9 energy landscape, it must be treated as position-dependent.

Mixing of free energies from different models: Varying experimental conditions can lead to significant differences in free energy values (hence the use of salt corrections in our work), and so we expect our Cas9 calculations to provide qualitative insights at best. In the limit that protein-NA interactions are so strong that sequence-dependent contributions to the free energy landscape are insignificant, Cas9-mediated R-loop formation may not be sequence-dependent, although existing experiments don't support this [8].

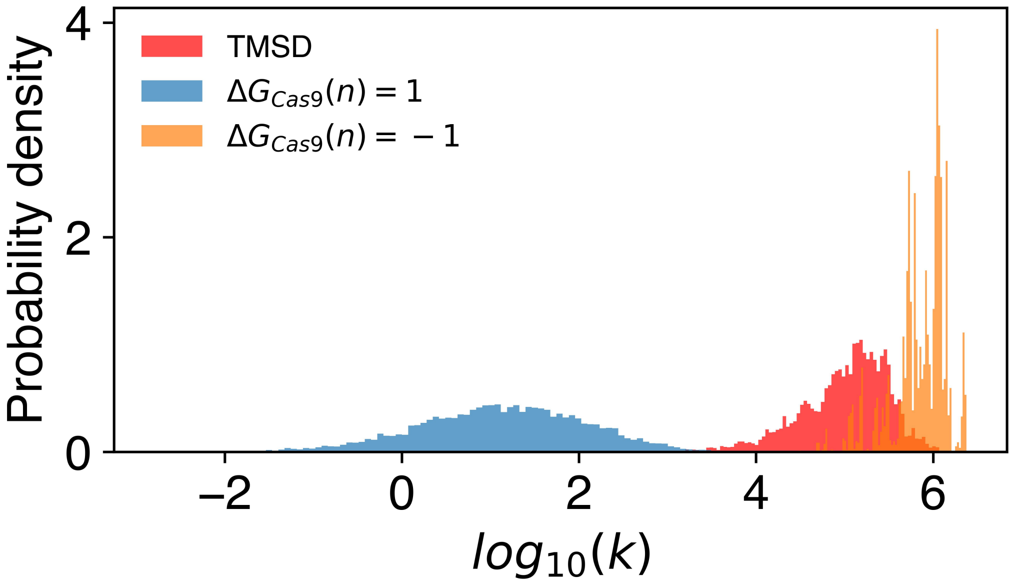

Supplementary Figure 5: Effect of different values of constant $\Delta G_{Cas9}(n)$ on rate distributions of random sequences, compared to regular TMSD, where $\Delta G_{Cas9}(n) = 0$.

Effect of different $\Delta G_{Cas9}(n)$ on the rate distribution: We observe that the rate distribution of random sequence pools widens when $\Delta G_{Cas9}(n)$ is included in the free energy profile, as discussed in the main text. However, the widening of the distribution doesn't depend on the details of $\Delta G_{Cas9}(n)$, and can still be observed if $\Delta G_{Cas9}(n)$ is simply made constant and positive, as shown in Supplementary Figure 4. Conversely, if $\Delta G_{Cas9}(n) = -1$, the distribution narrows. If $\Delta G_{Cas9}(n)$ is sufficiently large the distribution eventually narrows again, as the barrier becomes very difficult to overcome regardless of sequence.

Supplementary Note 9: Screening sequences for secondary structure

Unwanted interactions were carefully screened for, and for the vast majority of reactions studied, these are expected to have little to no effect on the observed dynamics. To screen for bimolecular interactions and secondary structure, the full sequence of each invader strand was entered into NUPACK [11], with temperature set to 20 degrees, strand concentration to 1 µM and size of the largest complex set to 3 strands. No multi-strand complexes are predicted to form, among both DNA and RNA invaders. The largest secondary structure free energy of any RNA invader was -0.67 kcal/mol, and for most sequences this free energy is 0.

Among DNA invaders, the largest free energy (in magnitude) was -2.1 kcal/mol (see Supplementary Figure 6), present in sequence 1b, and for that sequence we observe the largest deviation from the rate predicted by the kinetic model in DNA>DNA. That particular sequence was also the only one predicted to have stable base pairs in toehold nucleotides. The same DNA invader is also present in the DNA>RNA reaction, in which it displaces an RNA strand, and may slow down the observed kinetics of that reaction. However, for that particular case the effect of secondary structure is expected to be weaker, since the displacement free energy landscape is not flat. This is corroborated by the fact that deviation from the kinetic model prediction is not as great for reaction DNA>RNA as reaction DNA>DNA.

For all remaining DNA invaders, the largest secondary structure free energy is no less than -1.5 kcal/mol, and in those cases, the kinetic model predicts the reaction rates reasonably well.

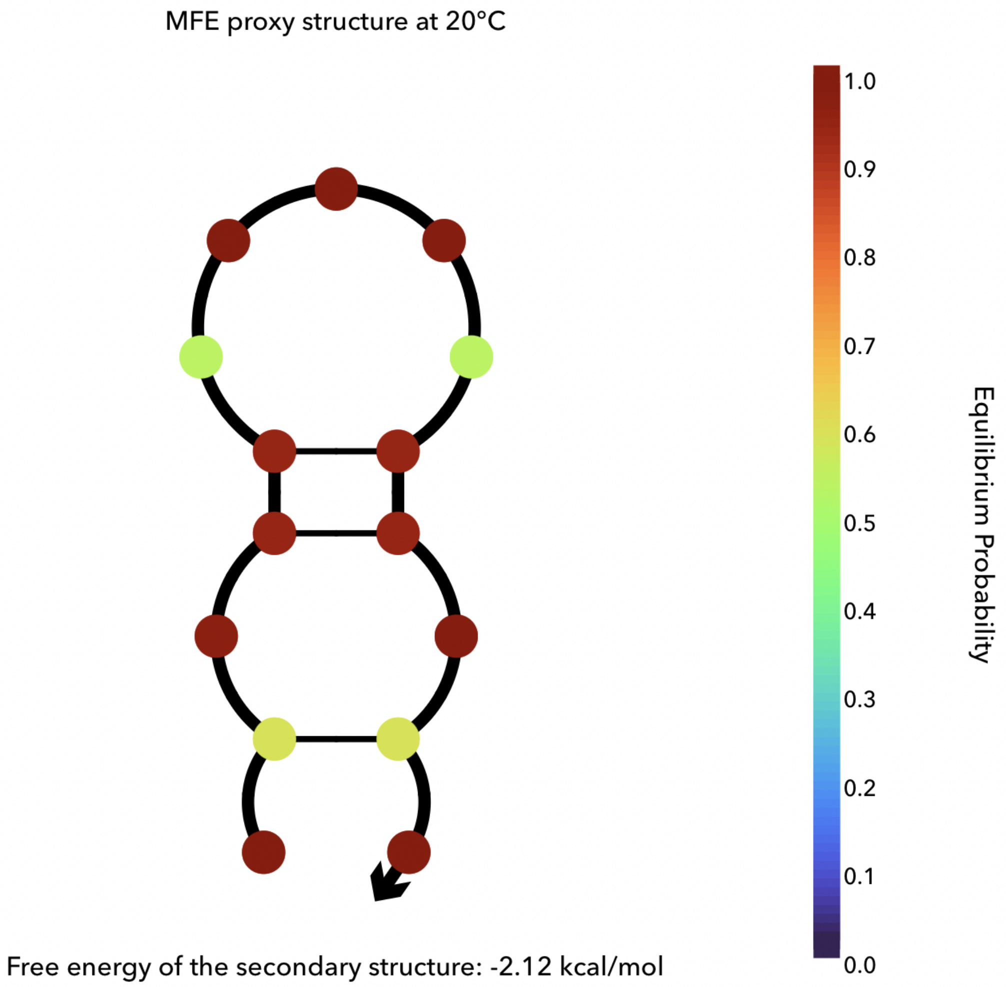

Supplementary Figure 6: Secondary structure of the DNA invader strand with sequence 1b. The same RNA sequence also has secondary structure but with an energy of −0.67 kcal/mol.

Supplementary References

[1] R. J. Allen, C. Valeriani, and P. Rein ten Wolde, *Forward flux sampling for rare event simulations*, Journal of Physics: Condensed Matter, 21 (2009), p. 463102.

[2] D. Banerjee, H. Tateishi-Karimata, T. Ohyama, S. Ghosh, T. Endoh, S. Takahashi, and N. Sugimoto, *Improved nearest-neighbor parameters for the stability of RNA/DNA hybrids under a physiological condition*, Nucleic Acids Research, 48 (2020), pp. 12042–12054.

[3] I. E. Ivanov, A. V. Wright, J. C. Cofsky, K. D. P. Aris, J. A. Doudna, and Z. Bryant, *Cas9 interrogates DNA in discrete steps modulated by mismatches and supercoiling*, Proceedings of the National Academy of Sciences, 117 (2020), pp. 5853–5860.

[4] A. Krueger, E. Protozanova, and M. D. Frank-Kamenetskii, *Sequence-dependent basepair opening in DNA double helix*, Biophysical Journal, 90 (2006), p. 3091â3099.

[5] S. Nakano, *Nucleic acid duplex stability: influence of base composition on cation effects*, Nucleic Acids Research, 27 (1999), pp. 2957–2965.

[6] N. Peyret, *Prediction of nucleic acid hybridization: Parameters and Algorithms*, PhD thesis, Wayne State University, January 2000.

[7] J. SantaLucia and D. Hicks, *The thermodynamics of DNA structural motifs*, Annual Review of Biophysics and Biomolecular Structure, 33 (2004), pp. 415–440.

[8] H. Shi, N. Al-Sayyad, K. M. Wasko, M. I. Trinidad, E. E. Doherty, K. Vohra, R. S. Boger, D. Colognori, J. C. Cofsky, P. Skopintsev, Z. Bryant, and J. A. Doudna, *Rapid two-step target capture ensures efficient CRISPR-Cas9-guided genome editing*, bioRxiv, (2024).

[9] F. G. Smith, J. P. Goertz, K. Jurinovic, M. M. Stevens, and T. E. Ouldridge, *Strong sequence dependence in RNA/DNA hybrid strand displacement kinetics*, Nanoscale, 16 (2024), pp. 17624–17637.

[10] N. Srinivas, T. E. Ouldridge, P. Sulc, J. M. Schaeffer, B. Yurke, A. A. Louis, J. P. K. Doye, and E. Winfree, *On the biophysics and kinetics of toehold-mediated DNA strand displacement*, Nucleic Acids Research, 41 (2013), p. 10641â10658.

[11] J. N. Zadeh, C. D. Steenberg, J. S. Bois, B. R. Wolfe, M. B. Pierce, A. R. Khan, R. M. Dirks, and N. A. Pierce, *Nupack: Analysis and design of nucleic acid systems*, Journal of Computational Chemistry, 32 (2010), pp. 170–173.
